# Methylome reorganization during *in vitro* dedifferentiation and regeneration of *Populus trichocarpa*

**DOI:** 10.1186/1471-2229-13-92

**Published:** 2013-06-25

**Authors:** Kelly Vining, Kyle R Pomraning, Larry J Wilhelm, Cathleen Ma, Matteo Pellegrini, Yanming Di, Todd C Mockler, Michael Freitag, Steven H Strauss

**Affiliations:** 1 Department of Forest Ecosystems and Society, 321 Richardson Hall, Corvallis, OR, USA; 2 Molecular and Cellular Biology Program, Corvallis, OR 97331, USA; 3 Department of Biochemistry and Biophysics, Corvallis, OR 97331, USA; 4 Oregon Health Sciences University, Portland, OR 97002, USA; 5 Department of Molecular, Cell and Developmental Biology, University of California, Los Angeles, CA 90095, USA; 6 Statistics Department, Oregon State University, Corvallis, Oregon, USA; 7 The Donald Danforth Plant Science Center, St. Louis, MO 63132, USA; 8 Center for Genome Research and Biocomputing, Oregon State University, Corvallis, OR 97331, USA

## Abstract

**Background:**

Cytosine DNA methylation (5mC) is an epigenetic modification that is important to genome stability and regulation of gene expression. Perturbations of 5mC have been implicated as a cause of phenotypic variation among plants regenerated through *in vitro* culture systems. However, the pattern of change in 5mC and its functional role with respect to gene expression, are poorly understood at the genome scale. A fuller understanding of how 5mC changes during *in vitro* manipulation may aid the development of methods for reducing or amplifying the mutagenic and epigenetic effects of *in vitro* culture and plant transformation.

**Results:**

We investigated the *in vitro* methylome of the model tree species *Populus trichocarpa* in a system that mimics routine methods for regeneration and plant transformation in the genus *Populus* (poplar). Using methylated DNA immunoprecipitation followed by high-throughput sequencing (MeDIP-seq), we compared the methylomes of internode stem segments from micropropagated explants, dedifferentiated calli, and internodes from regenerated plants. We found that more than half (56%) of the methylated portion of the genome appeared to be differentially methylated among the three tissue types. Surprisingly, gene promoter methylation varied little among tissues, however, the percentage of body-methylated genes increased from 9% to 14% between explants and callus tissue, then decreased to 8% in regenerated internodes. Forty-five percent of differentially-methylated genes underwent transient methylation, becoming methylated in calli, and demethylated in regenerants. These genes were more frequent in chromosomal regions with higher gene density. Comparisons with an expression microarray dataset showed that genes methylated at both promoters and gene bodies had lower expression than genes that were unmethylated or only promoter-methylated in all three tissues. Four types of abundant transposable elements showed their highest levels of 5mC in regenerated internodes.

**Conclusions:**

DNA methylation varies in a highly gene- and chromosome-differential manner during *in vitro* differentiation and regeneration. 5mC in redifferentiated tissues was not reset to that in original explants during the study period. Hypermethylation of gene bodies in dedifferentiated cells did not interfere with transcription, and may serve a protective role against activation of abundant transposable elements.

## Background

A growing body of evidence documents extensive epigenetic changes as a result of *in vitro* plant tissue culture [1]. The genetic and epigenetic mutations induced can be a detriment to clonal propagation but they can also provide a tool for producing stress-tolerant and/or disease resistant plants by *in vitro* selection of somaclonal variants [2,3]. However, the nature of the epigenetic changes produced by *in vitro* regeneration are poorly known, particularly on a genome scale.

The process of eukaryotic cellular dedifferentiation is often referred to as a return to a ‘stem-cell like’ state, as cells first must re-enter the cell cycle. This developmental shift involves large-scale chromatin reorganization, leading to acquisition of pluripotency (reviewed by [4]). Cellular differentiation, in contrast, occurs in response to the balance of growth regulators in the culture medium and ultimately leads to organogenesis. This transition involves cell fate decisions and eventual exit from the cell cycle. Both dedifferentiation and differentiation involve changes in expression of key genes. Epigenomic reprogramming underlying these large developmental shifts is thought to be a major cause of somaclonal variation.

Somaclonal variation can be a serious problem in commercial nurseries, occurring both in the field and during *in vitro* propagation. While the intention of clonal propagation is regeneration of phenotypically identical individuals, it is often not the case in practice. This is illustrated by the *mantled* floral phenotype in oil palm (*Elaeisguineensis* Jacq.), which affects ~5% of regenerated palms [5]. The *mantled* mutation results in abnormal flowers, fruits, and ultimately decreased oil yield. Several studies have revealed genome-wide DNA hypomethylation in mantled somaclones compared to normal counterparts [6-8], demonstrating that changes in DNA methylation can be associated with phenotypes occurring after *in vitro* propagation.

DNA methylation is known to vary as a result of *in vitro* culture in other species as well. Methylation-sensitive amplified polymorphism (MSAP) has been widely usedto study epigenetic instability during regeneration [2,9-11]. Comparisons of banding patterns between regenerants and donor plants in *Freesia*, oil palm and hops (*Humulus lupulus* L.) generally show both gains and losses of polymorphic bands, but substantially higher proportions of polymorphisms showing demethylation at the target site [9,11,12]). Using the MSAP method, hop plants from repeated rounds of regeneration from callus tissue were found to have increased 5mC differentiation compared to donor tissue [11]. In cocoa (*Theobroma cacao* L.), MSAP variability in regenerants from leaves and staminodes increased over time in culture [13].

Changes in chromatin structure have been observed during *in vitro* dedifferentiation, and have mainly been studied in *Arabidopsis* protoplast systems. On *Arabidopsis* chromosome I, changes included condensation of 18 s ribosomal DNA and decondensation of telomeric and pericentromeric regions, but no changes in centromeric repeats [14]. In long-term *Arabidopsis* suspension cultures, examination of chromosome IV showed that methylation of euchromatin increased and methylation of heterochromatin decreased [15]. Studies of genomic 5mC content of *Arabidopsis* dedifferentiating cells have reported mixed results. Elhiti et al. [16] observed global DNA hypomethylation during the induction phase of somatic embryogenesis in *Arabidopsis*, but Tessadori et al. [17] reported no difference in genomic 5mC during chromatin decondensation in *Arabidopsis* mesophyll cells dedifferentiating into protoplasts. It appears that epigenomic changes vary widely among chromatin domains, and vary widely among the cell types and culture systems employed.

In general, plant transposable elements are transcriptionally inactivated by cytosine methylation [18]. Under *in vitro* culture conditions, methylation of specific families of transposable elements is altered and demethylation can result in transcriptional reactivation [19,20]). In oil palm, transposable elements are generally hypomethylated at the dedifferentiated stage then remethylated in regenerated plants, but to a lower level than donor plants [21].The 5’ LTR portion of an LTR retroelement of the *Gypsy* superfamily *LORE1* was demethylated and in some cases transcriptionally activated in regenerated plants of *Lotus japonicus*[19]. In addition, this LTR-*Gypsy* retroelement was transpositionally active in pollen of regenerated plants. Comparisons of active and inactive copies of the MITE element *mPing* in rice (*Oryza sativa* L.) calli and regenerated plants showed hypermethyation of flanking regions of the immobile copy [22]. Comparisons of 5mC in Arabidopsis long-term suspension cultures relative to leaf cells showed that transposable elements in heterochromatin were hypomethylated, including *Athila* and LTR-*Copia* retroelements and *AtMu* DNA transposons. However, LTR-*Gypsy* class retrotransposons had become hypermethylated [15]. Variation among transposon classes in their epigenetic responses to *in vitro* culture may be an important source of the variation observed among chromatin domains.

Methylation of particular genes has been shown to change during *in vitro* plant dedifferentiation and redifferentiation. In *Arabidopsis*, cellular dedifferentiation was accompanied by MET1- and DRM2-mediated promoter hypermethylation of *MAPK12*, *GSTU10* and *BXL1* in callus cells, and of *TTG1*, *GSTF5*, *SUVH8*, *fimbrin* and *CCD7* in cell suspension cultures [23]. Transcription of genes involved in DNA methylation, including *met1*, *cmt3*, *drm1* and *drm2* were upregulated in *Arabidopsis* cell suspension culture, while the DNA demethylase ros1 was downregulated [15].

There have been few studies of genome-scale reorganization during *in vitro* organogenesis, and most have focused on the transcriptome. In rice (*Oryza sativa*), only one to three percent of genes showed differential regulation during organogenesis of shoot, roots, and somatic embryos [24]. Bao et al. [25] studied transcriptome changes in a *Populus tremula*x *P. alba* hybrid during *in vitro* dedifferentiation and organogenic regeneration using a commercial (Affymetrix) oligonucleotide genome-scale microarray. In sequential time-point comparisons, the greatest number of differentially-regulated genes (~9,000) was detected at the early callogenesis stage, with roughly equal numbers of upregulated and downregulated genes. They identified a large number of overrepresented Gene Ontology (GO) categories associated with the transition, including those related to protein metabolism, stress response, cellular signaling, and transcription. Surprisingly, there were far fewer genes that were differentially regulated during shoot regeneration from callus. In the same poplar system, Bao et al. [26] showed that regulatory genes in meristem development, including members of the *WUSCHEL (WUS)* and *SHOOTMERISTEMLESS (STM)* families, were among the genes downregulated at early callus initiation, and that one of the two poplar *WUS* paralogs was upregulated during shoot initiation, though the other was not differentially regulated.These studies provide further evidence of the highly complex and variable nature of gene expression programs during *in vitro* regeneration.

To further understanding of epigenetic modification and its relationship to transcription in poplar, we compared genome-wide 5mC profiles in stem internode tissues at three stages of *in vitro* plant regeneration: the micropropagated internode explant, undifferentiated callus, and internodes from regenerated shoots while still *in vitro*. To facilitate genome sequence mapping, we used genotype ‘Nisqually-1’ of *Populus trichocarpa,* from which the *Populus* reference genome sequence was derived [27]. We analyzed changes as 1 kb tiled genome windows throughout the genome, as well as specific genome features including gene promoters, gene bodies, and transposable elements. In addition, we compared 5mC profiles among the three tissue culture stages to the *Populus* Affymetrix microarray dataset [25] to ascertain if any broad relationships between cytosine methylation and gene expression exist. We report substantial changes in DNA methylation during regeneration, changes in methylation that varied widely among chromosomal and genic regions, and relationships of methylation in specific genic regions to gene expression.

## Results

### Sequencing samples and MeDIP-seq read mapping

**Figure 1:**
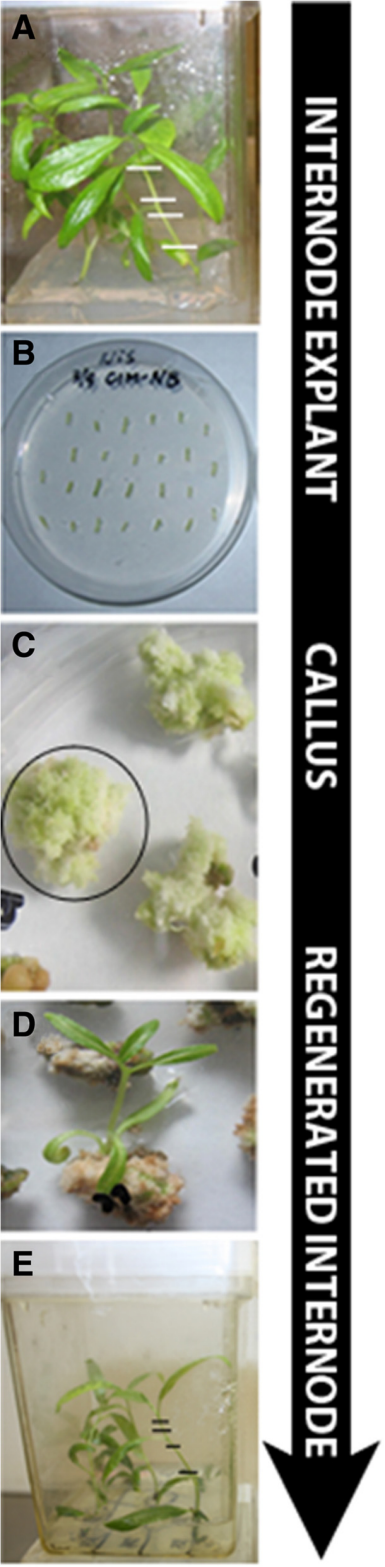
**Plant materials used in this study.** All *in vitro* materials were derived from the *P. trichocarpa* Nisqually-N1 genotype. **A**. Starting material. Internodes (shown with hash marks) were cut from thirty-eight day old Nisqually *In vitro* plants grown on Woody Plant Medium (WPM). **B**. Stem explants consisted of internodes 3–5 cm long; these were cultured on Callus Induction Medium (CIM-NB) in darkness for 4 weeks. **C**. Large calli formed on CIM-NB medium in the dark after 4 weeks of culture; whole calli (indicated by circle) were sampled. **D**. Remaining calli were transferred to Shoot Induction Medium (SIM-BN), where they were kept in light and were sub-cultured every two weeks. **E**. Shoots regenerated after 75 days of culture. Internodes (shown with hash marks) were excised from regenerated stems.

Tissues were sampled from three *in vitro* culture stages: internode explants, four-week-old calli derived from the explants, and internodes from stems regenerated from the calli (Figure 1). Each sample included three biological replicates. Three to six lanes of Illumina MeDIP-seq data were obtained for each sample (Table 1). A non-immunoprecipitated control sample was also sequenced.

**Table 1:** Summary of sequencing samples and results

**Tissue**	**Biological**	**Illumina GAll**	**Total reads**	**Mapped reads**	
	**Replicates**	**Lanes sequenced**		**No**	**%**
Internode explant	3	6	204, 768, 821	84,814, 829	41.4
Callus	3	6	214, 470, 025	48, 813, 758	22,8
Regenerated internode	3	3	147, 824, 747	35, 116, 532	23.8
Input	4	4	61, 453, 962		
**Totals**	**13**	**19**	**628, 517, 555**	**168,745, 119**	

Sequencing was performed on an Illumina GAIIx platform. Mapped reads include all non-clonal reads that aligned to unique loci on the reference genome, allowing up to two mismatches.

**Figure 2:**
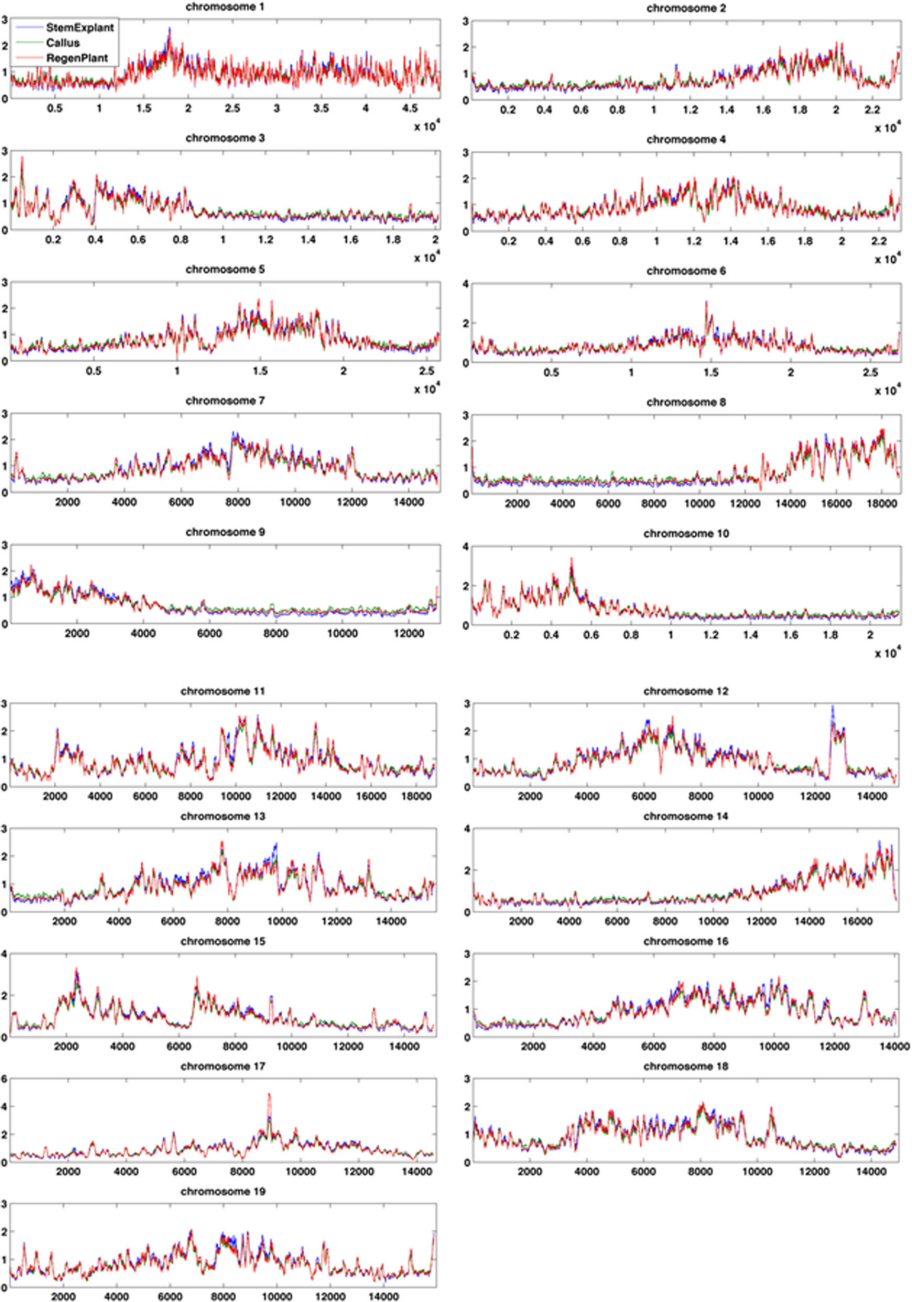
**Distribution of methylation along chromosomes.** MeDIP-seq read counts were plotted in 1 kb windows, with chromosomes scaled to a uniform width. One line, the average over biological replications, is shown for each tissue type.

Sequencing reads were plotted in 1 kb tiled genome windows along *P. trichocarpa*‘s 19 chromosomes, revealing highly heterogeneous 5mC profiles that were broadly consistent among the tissues (Figure 2). However, on several chromosomes with long stretches of relatively low read depth (e.g. left half of chromosome 8, right halves of chromosomes 9 and 10), callus tissue appeared to have a moderately higher read depth than either explant or regenerated tissues.

Reads per 1 kb window per million mapped reads (RPKM) values were calculated for each biological replicate within tissues (Figure 2). Mean RPKM variance among biological replicate samples per window ranged from 4.5-9.3 for explants, 5.1-8.6 for calli, and 6.1-34.9 for regenerated internodes. When biological replicates for all samples were subjected to hierarchical clustering analysis using RPKM calculations over all annotated gene bodies, non-immunoprecipitated control samples formed a distinct cluster, and explant and callus tissues formed a separate cluster (Additional file 1). One of the three regenerated internode replicates clustered with explant and callus replicates, while the other two regenerated internode replicates clustered separately.

### Decrease in overall genome 5mC during in vitro culture

**Figure 3:**
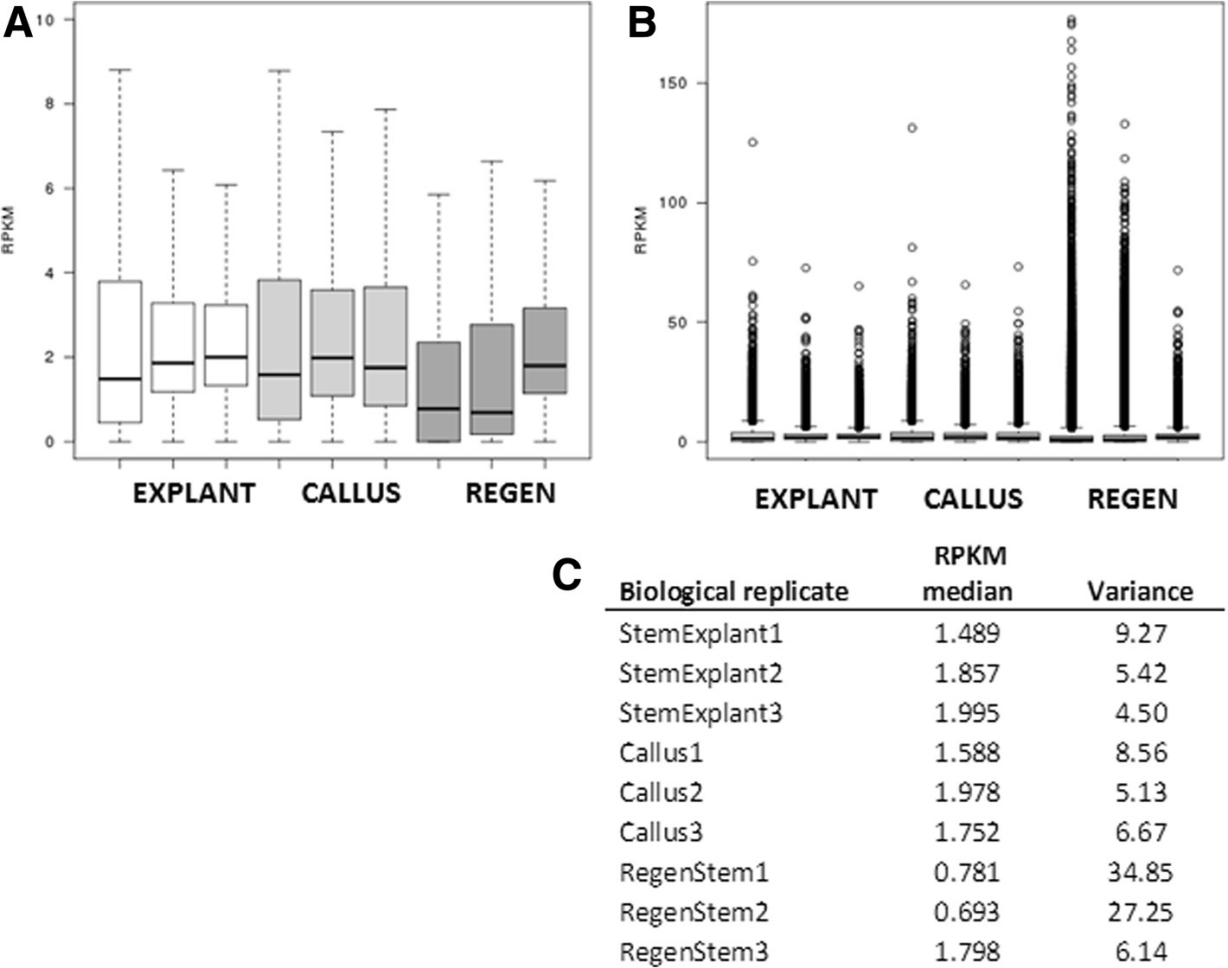
**RPKM range for 1 kb tiled genome windows, by biological replicate.** For box plots, each box encloses the middle 50% of the distribution (25th percentile – 75th percentile, or interquartile range (IQR)). Lines in boxes mark medians. Lines extending from boxes mark minimum and maximum values that fall within 1.5 times the IQR. **A**. Outlier points excluded. **B**. Outlier points included. **C**. Means and variances for each biological replicate.

Cytosine DNA methylation was determined to be enriched in 1 kb tiled genome windows compared to the non-immunoprecipitated control sample using two methods: RPKM at a 1% false discovery rate (FDR), and a negative binomial method at 10% and 20% FDR. The negative binomial method included additional pairwise comparisons among the three stages. Pooling data across tissues, agreement between the methods was 51.5% for 5mC-enriched 1 kb tiled genome windows and 82.5% for non-enriched windows (FDR of 10% for negative binomial analysis: Table 2). When tissues were evaluated individually, the two methods agreed for 55.4% of 5mC-enriched window calls for explants, 54.2% for callus, and 58.9% for regenerated internodes (Table 3). RPKM calculations for individual biological replicates for each tissue showed substantially higher variance in regenerated internodes than in explants or calli; two of three biological replicates had variances two- to three-fold above those of the other tissues (Figure 3).

**Table 2:** Genome methylation tallied over 1 kb tiled genome windows, with tissues pooled

**No. methylated 1 kb windows**			
**All tissues**	**RPKM**	**NB**	**Common**
**Methylated**	33, 537	56, 861	30,727 (51.57%)
**Unmethylated**	261, 452	267, 876	239, 225 (82.5%)
**Tissue specific**	33, 921	44, 910	18, 755(31.2%)
**Tissue-specific proportion**	0,50	0.44	

Numbers are counts of windows called methylated compared to non-immunoprecipitated control by one of two methods: *RPKM* at a 1% false discovery rate, or negative binomial analysis (FDR of 10%). The *P. trichocarpa* genome is comprised of 378,536 windows. Percentage of common windows was calculated by dividing the total number of windows called by both methods by the total of those called by either method.

**Table 3:** Genome methylation tallied over 1 kb tiled genome windows, by tissue

**No. methylated 1 kb windows**			
**By tissue**	**RPKM**	**NB**	**Common**
**Internode explant**	55, 036	92, 401	52, 566 (55.4%)
**Callus**	51, 886	81, 444	46, 863 (54.2%)
**Regen internode**	42, 511	63, 722	39, 384 (58.9%)

Numbers are counts of windows called methylated compared to non-immunoprecipitated control by one of two methods: *RPKM* at a 1% false discovery rate, or negative binomial analysis (FDR of 10%). The *P. trichocarpa* genome is comprised of 378,536 windows. Percentage of common windows was calculated by dividing the total number of windows called by both methods by the total of those called by either method.

**Figure 4:**
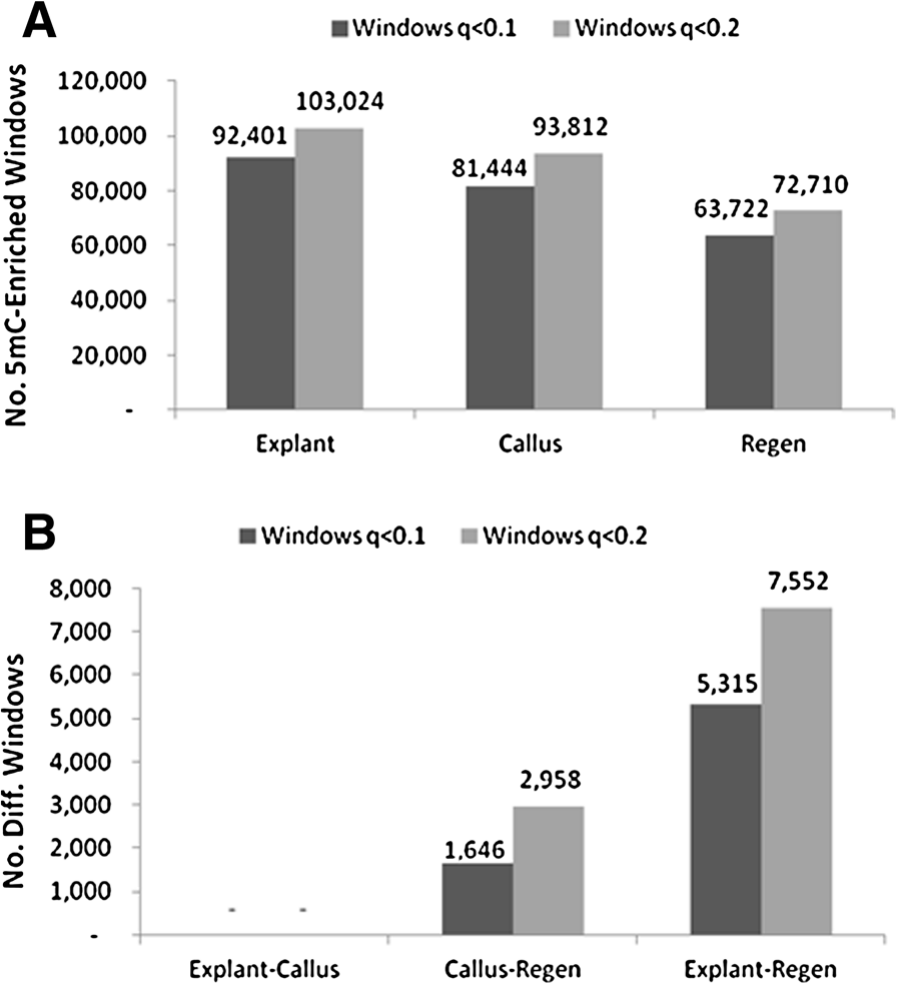
**Genome methylation of in vitro tissues based on 1 kb tiled genome windows. A)** Windows were called mC-enriched in MeDIP-seq samples compared to a sequenced non-immunoprecipitated control sample at two FDR levels. **B)** Number of windows with different 5mC enrichment in pairwise comparisons at two significance levels.

Based on negative binomial results, 26.9% of the genome was enriched in 5mC in all tissues, and 73.1% was non-enriched. Of the 5mC-enriched fraction, 64.2% was differentially-enriched among internode explants, calli, and regenerated internodes, while 35.8% was 5mC-enriched in all three tissues. The number of 5mC-enriched windows was lower in calli (21.5%) than in explants (24.4%) and lower still in regenerants (16.8%) (Figure 4). In pairwise comparisons, explants and calli were not significantly different at any windows, but regenerants differed from calli at 1,646 to 2,958 windows, and the number of significantly different windows between regenerants and explants was approximately three-fold greater than this.

### Gene body 5mC changes were different from those of gene promoters and intergenic space

**Figure 5:**
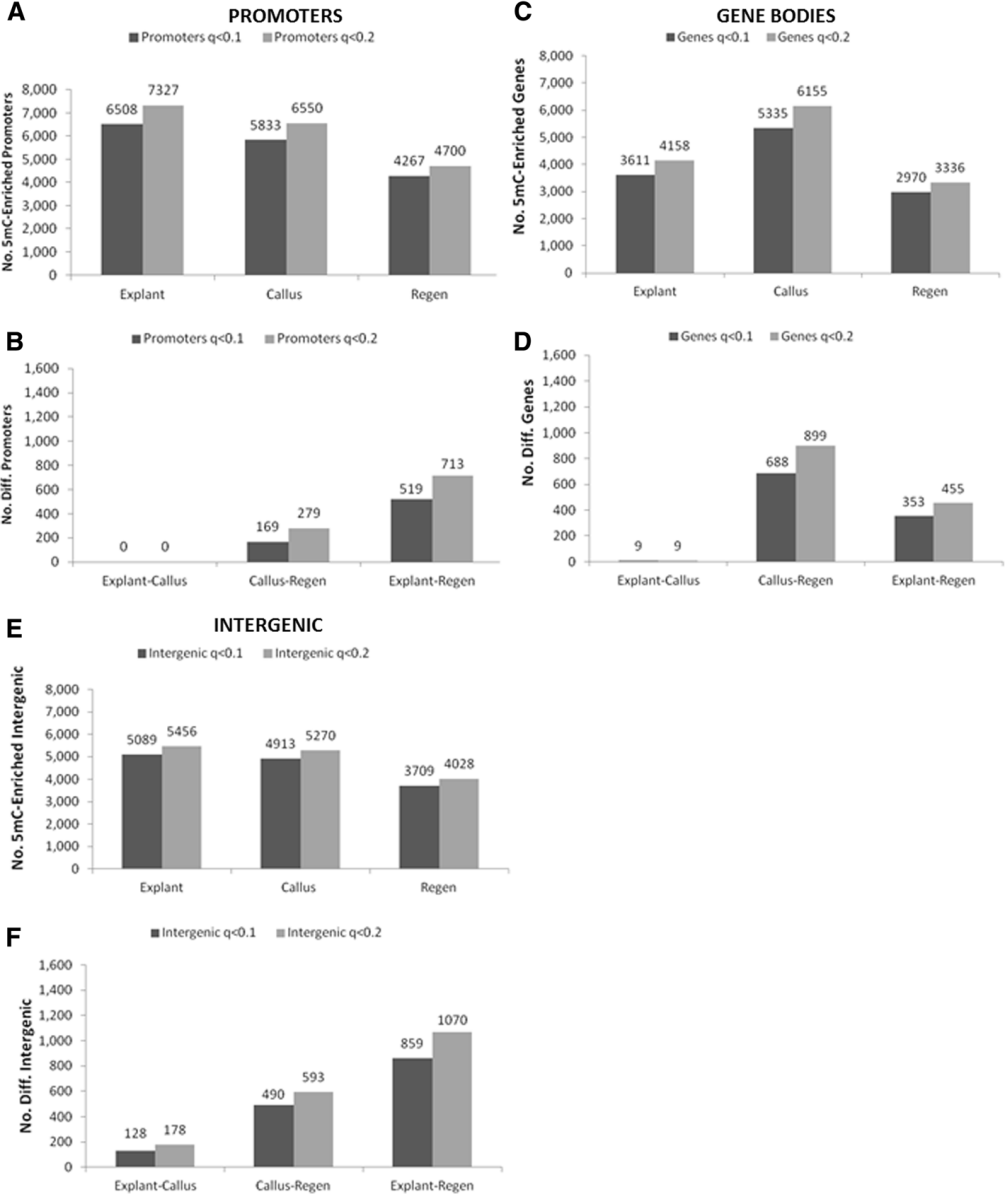
**Variation in 5mC among gene features.** Numbers of 5mC-enriched promoters, 5’ and 3’ UTRs, gene bodies, and intergenic spaces were determined compared to a non-immunoprecipitated control sample at two levels of significance . **A**. Gene promoters. **B**. Gene promoter pairwise comparisons. **C**. Gene bodies **D**. Gene bodies pairwise comparisons. **E**. Intergenic spaces. **F**. Intergenic spaces pairwise comparisons.

**Figure 6:**
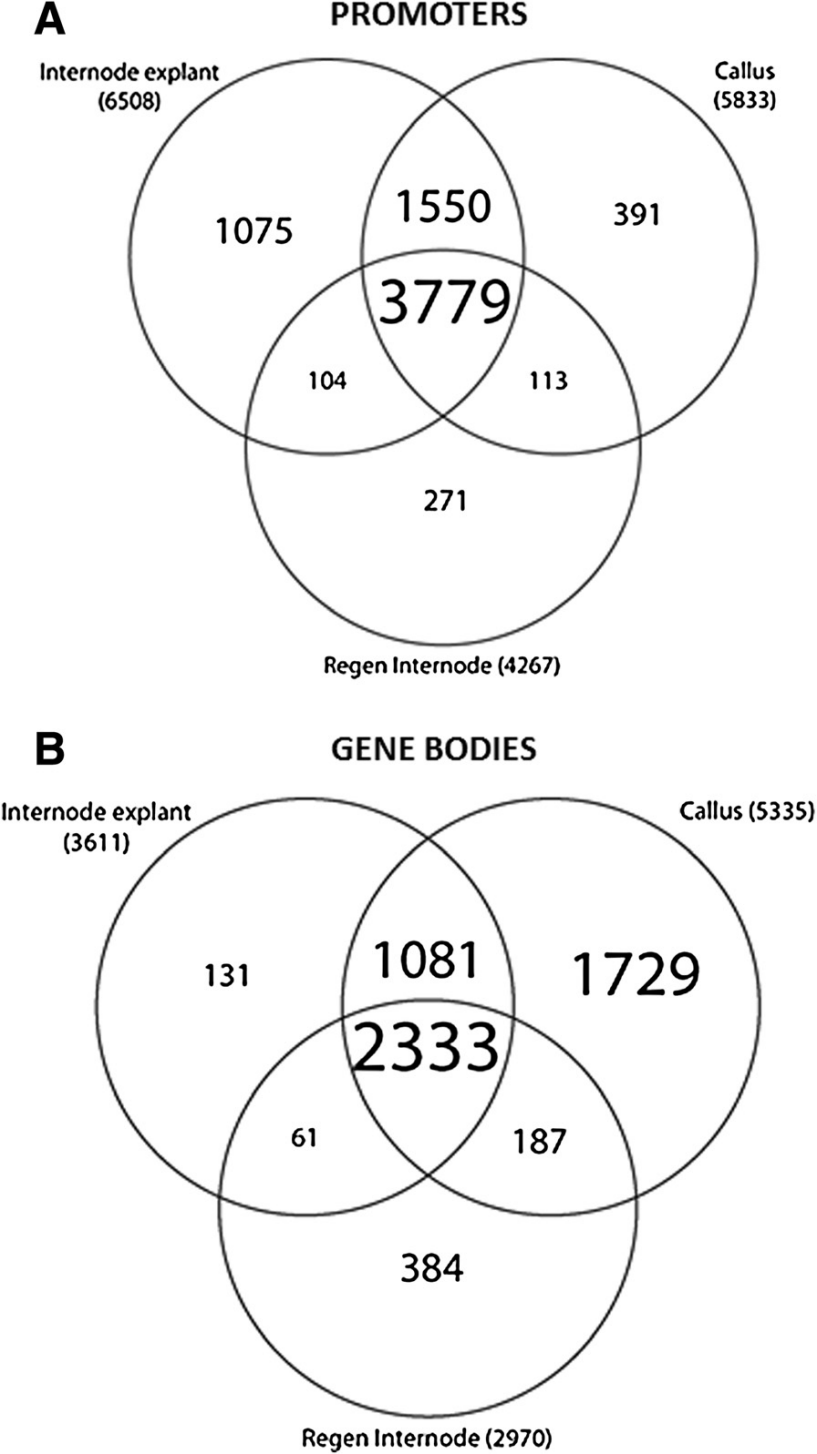
**Differentiation of promoter and gene body methylation among tissues.** Venn diagrams show number of genes with common promoter or gene body methylation among the sampled tissue types. Numbers are counts of genes called methylated compared to input (q < 0.1). Numbers in parentheses are total counts of methylated genes at that gene feature for that tissue type. **A**. Promoters. **B**. Gene bodies.

**Figure 7:**
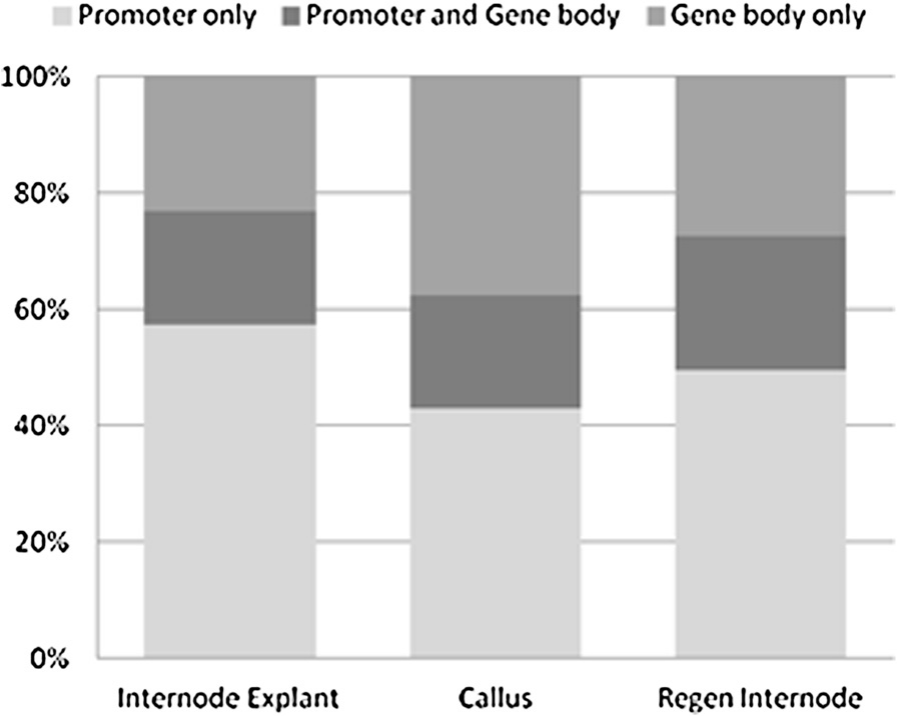
**Overlap of promoter and gene body methylation within tissues.** Relative proportion of genes with 5mC enrichment at promoters, gene bodies, or both is shown for each tissue. Numbers based on gene-associated features called methylated compared to input (q < 0.1).

Gene promoter 5mC showed a decrease during *in vitro* culture that was similar among tissues as that of the genome as a whole. About 17% of gene promoters were 5mC-enriched in explants, whereas 15% were enriched in calli and 11% were enriched in regenerants (Figure 5). Pairwise differences also showed the greatest difference between explants and regenerated internodes. Intergenic 5mC showed a very similar trend to that of promoters, though with approximately 20% fewer windows called significant. Of the 7,283 promoters enriched in 5mC, 3,779 (52%) were enriched at all three stages and 48% showed tissue differentiation (Figure 6). Of 5mC-enriched genes, 43-57% were enriched in promoters only, while 19-23% were enriched at both features (Figure 7).

Gene bodies – which consist of annotated gene transcripts – had a very different pattern of methylation than promoters or intergenic DNA. Over 60% (3,573 of 5,906) of body-methylated genes showed tissue differentiation (Figure 7), mainly because gene body 5mC was markedly higher in callus (Additional file 2). Of the 3,573 body-methylated genes with tissue differentiation, 1,729 (48%) were transiently methylated (methylated during tissue dedifferentiation, then demethylated during organogenic regeneration). In contrast, only 61 genes (1%) showed the opposite pattern of demethylation during dedifferentiation and remethylation during regeneration. Relatively few untranslated regions were 5mC-enriched. Only 0.3% to 1% of 5’UTRs were enriched, with none significantly different in pairwise comparisons.Only 1% to 3% 3’of UTRs were 5mC-enriched, with less than 10 significantly different in pairwise comparisons.

### Transposable elements show 5mC increase during in vitro culture

Four major transposable element categories encompassing ~10% of the poplar genome were examined for 5mC-enrichment. A methylation index was calculated for each category that accounted for the number of basepairs in each category (Table 4). In all three of the four categories, the methylation index was higher in calli relative to explants, and higher in regenerated internodes relative to calli. The *Enspm* category did not have any 5mC-enriched elements in either explants or calli, but did in regenerated internodes.

**Table 4:** Transposable element methylation increases through dedifferentiation and organogenesis for abundant elements

**Type**	**Explant**	**Callus 5mc**	**Regen 5mC**
	**5mC Index**	**Index**	**Index**
**Copia**	65,660	193, 331	500, 473
**Enspm**	-	-	112, 738
**Gypsy**	130, 587	152, 883	499, 257
**Ogre**	8, 482	21, 455	65, 362

Significant methylation enrichment was determined using the negative binomial method. Methylation index was calculated by dividing the number of methylation-enriched elements in each category by the total number in the genome, then multiplying this quantity by the total length in base pairs of all elements in that category.

### Gene body methylation associated with GO enrichmentand gene density

To identify biological processes most likely to be undergoing epigenetic perturbations during dedifferentiation, we conducted a Gene Ontology (GO) enrichment analysis of genes that underwent transient body hypermethylation. We found that 189 distinct GO categories were overrepresented. The most highly significantly enriched GO categories included those related to protein cellular localization/intracellular transport/catabolism, DNA and RNA metabolism, and signal transduction (Table 5, Additional file 3).

**Table 5:** Overrepresented Gene Ontology (GO) categories of genes with transient body methylation

**GO term**	**Ontology description**		**Number in input list**	**Number in BG/Ref**	**p-value**	**FDR**
**Protein metabolism**						
GO: 0006886	P	Intracellular protein transport	32	187	8.10E-13	1. 70E-10
GO: 0034613	P	Cellular protein localization	33	199	8.90e-13	1. 90E-10
GO: 0030163	P	protein cataolic process	19	156	6.50E	0.0011
GO: 00065111	P	ubiquitin-dependent protein catabolic process modification-dependent protein catabolic	17	130	7.50E-06	0.0012
GO: 0019941	P	process	17	130	7.50E-06	0.0012
**Nucleic acid metabolism**						
		nucleobase, nucleoside and nucleotide				
GO: 0055086	P	metabloic process	28	256	4.80E-07	9.20E-05
GO: 0006259	P	DNA metabolic process	25	214	5.60E-07	0.00011
Go: 0016070	P	RNA metabloic process	87	1366	2.10E-06	0.00037
GO: 0034660	P	nCRNA metablic process	24	141	5.90E-10	1.20E-07
GO: 0006396	P	RNA processing	22	141	3.00E-06	0.00053
GO: 0006399	P	tRNA metabloic process	20	108	3.30E-09	6.60E-07
**Cellular signalling**						
regulation of small GTPase mediated signal						
Go: 0051056	P	transduction	13	63	4.80E-07	9.20E-05
GO: 0046578	P	regulation of Ras protein signal transduction	13	63	4.80E-07	9.20E-05
GO: 0007265	P	Ras protein signal transduction	13	63	4.80E-07	9.20E-05
GO: 0009966	P	regulation of signal transduction	13	67	1.00E-07	0.00018
GO: 0023051	P	regulation of signaling process	13	67	1.00E-06	0.00018
GO: 0010646	P	regulation of cell communication	13	37	1.00E-06	0.00018
GO: 0032011	P	ARF protein signal transduction	7	34	0.00022	0.029

Genes were determined to be methylated at their gene bodies compared to a non-immunoprecipitated control using a negative binomial analysis (q < 0.1). GO analysis was performed with the AgriGo Singular Enrichment Analysis tool (http://bioinfo.cau.edu.cn/agriGO/analysis.php). Categories shown are a subset of the total 189 significantly enriched (p < 0.05) categories, all of which are shown in Additional file 3.

**Figure 8:**
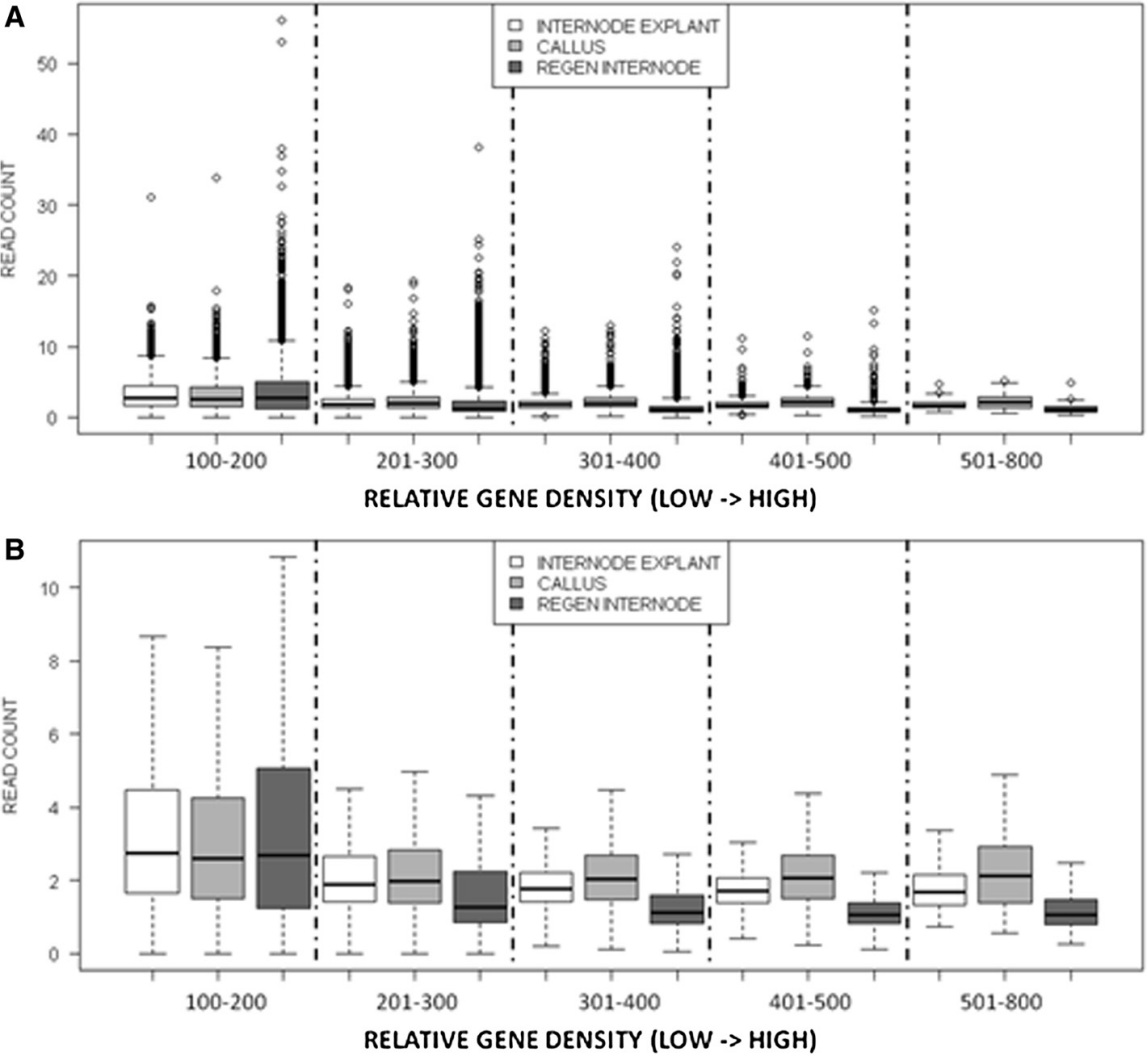
**Boxplots showing 5mC quantity (read count, y axis) in 10 kb tiled windows.** Gene density was ranked on a scale from 100 to 800. **A**. Including outliers. **B**. With only 50th percentile boxes shown.

**Figure 9:**
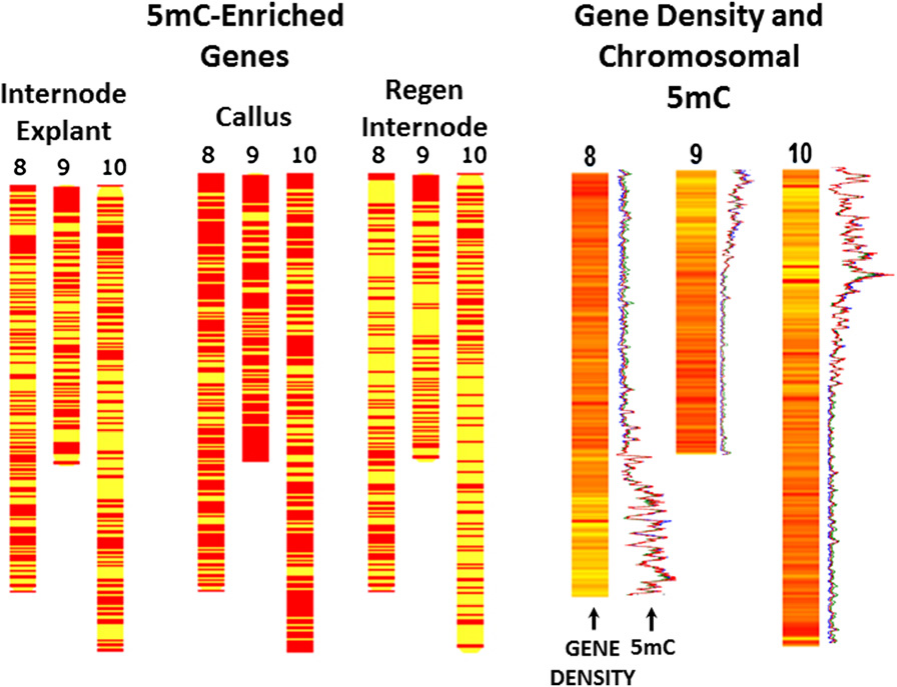
**Relationship of chromosomal gene density on chromosomes 8, 9, and 10 to 5mC changes during *in vitro *regeneration.** Genes with methylated gene bodies (q < 0.1) are plotted on *P. trichocarpa* chromosomes 8, 9, and 10. Gene density is shown on the same chromosomes to the right, where darker areas have higher gene density. MeDIP-seq 1 kb window plots from Figure 2 are shown to the right of each chromosome.

The relationship between body-5mC-enriched genes and gene density along chromosomes was compared among *in vitro* development stages. When gene density was ranked and organized into five bins from low to high,chromosomal regions with the lowest gene density had the highest number of 5mC-enriched gene bodies (Figure 8A-B); this was true for all three stages. Pearson’s correlation coefficient was −0.36 for explants, -0.26 for calli, and −0.38 for regenerated internodes (p < 0.001). When all tissues were pooled, the correlation was −0.35 (p < 0.001). In all but the lowest gene density bin, calli had a significantly higher gene body 5mC enrichment than either explants or regenerated internodes (paired t-tests, p < 0.001). Chromosomal regions with higher gene density contained genes that became hypermethylated in callus, then demethylated in regenerated internodes.This trend can be visualized with reference to chromosomes 8–10, three chromosomes with distinct regions of low gene density and high methylation (Figure 9). The chromosomal regions of high gene density all showed a prominent increase in 5mC in callus compared to explants and then a decrease from callus to regenerants.

### Association of methylation and gene expression

Genic 5mC enrichment was compared to levels of gene expression using previously-obtained Affymetrix microarray data. When gene expression was divided into deciles from low to high, genes with intermediate expression exhibited the highest median 5mC levels, and this trend was consistent for all tissues (Additional file 4). However, when mean 5mC levels were considered, the tissues had distinct expression/5mC profiles (Additional file 5). In all tissues, a near bell-shaped curve was observed, where genes with intermediate expression levels were higher in 5mC than weakly or highly expressed genes. At nearly all expression levels, calli had a higher median RPKM and greater RPKM range than did either explants or regenerated internodes (Additional file 4).

We compared expression levels of sets of genes with different 5mC status. Expression of genes with 5mC-enriched promoters did not differ significantly from unmethylated genes (Additional file 6). However, median expression of genes with 5mC-enrichment in both promoters and gene bodies was substantially lower than that of non-5mC-enriched genes in all tissues. In explants and regenerated internodes, genes that were 5mC-enriched in gene bodies and not in promoters had significantly lower expression than genes 5mC-enriched in both promoters and gene bodies (p < 0.05). In contrast, genes in this category in calli had the highest median expression of genes in any category in any tissue.

We examined genes related to hormone signaling including auxin, abscisic acid (ABA) and gibberellic acid (GA) signaling, as well as six categories of transcription factors related to stress response, many of which were differentially regulated during *in vitro* culture [25]. In all cases, 5mC increased in callus tissue relative to explants, then decreased in regenerated internodes, but there was no clear relationship between RPKM and gene expression (data not shown).

### Expression of DNA methyltransferases

To probe the causes of changes in 5mC among tissues, we examined the expression of annotated methyltransferase homologs. Of nine homologs of Arabidopsis *DNA methyltransferase (MET1/2*), *CHROMOMETHYLASE 3 (CMT3)*, *DECREASE IN DNA METHYLATION 1 (DDM1)*, and *DOMAINS REARRANGED METHYLTRANSFERASE 1* and *2 (DRM1/2)*, three showed >1.5-fold expression changes during dedifferentiation and redifferentiation: POPTR_0019s00240 (*MET1*), POPTR_0004s14140 (*MET2*), and POPTR_0007s12710 (*DDM1*). Each showed an upward trend through both callus and regenerated internode stages (Additional file 7). Expression of one of the three *DRM1/2* homologs (POPTR_0010s16200) showed a slight downward trend. Expression of the second *DDM1* homolog (POPTR_0019s15030) showed a slight upward trend. Expression of the two *CMT3* homologs (POPTR_0003s21520, POPTR_0003s21510), and two of the three *DRM1/2* homologs (POPTR_0001s35160, POPTR_0014s04840), did not change appreciably (<50%).

## Discussion

*In vitro* plant regeneration is an inherently complex process involving large-scale developmental reprogramming. To begin to identify the epigenomic marks important to regulation of *in vitro* cellular dedifferentiation and organogenic regeneration in poplar, we compared cytosine DNA methylation profiles from internode explant, callus, and regenerated internode stages. In previous work, by bisulfite sequencing of diverse targets we showed that the MeDIP-seq method gives an accurate estimate of 5mC in poplar tissues [28]. A number of surprising and highly diverse trends were found, which are summarized in Table 6.

**Table 6:** **Summary of changes in methylation during *in vitro *development for genome features**

**FEATURE**	**Explant to callus**	**Callus to regen**
Whole genome (tiled windows)	+ 12%	- 22%
Intergenic spaces	- 3%	- 24%
Gene promoters	- 10%	- 27%
5’UTRs	- 48%	+ 324%
3’ UTRs	+ 8%	+ 86%
Gene bodies	+ 47%	- 44%
Transposable elements	+ 80%	+ 240%

Data is changes in percent of genes called methylated by the negative binomial method at a false discovery rate of 10%. Calculated as: 100*[(Number of genes called in tissue 1 – number called in tissue 2) / mean number of genes called in tissue 1].

Although we found some strong associations between expression and 5mC, we believe that these are minimal estimates of the strength and diversity of these associations. First, our work was done with a *P. trichocarpa* genotype that was not the same species as the *P. tremula x tremuloides* genotype that had been used for expression profiling. Second, only 27,753 of the genes represented on the expression array, which was based on the v.1.1 poplar genome assembly, could be cross-referenced to the 39,009 genes in the v2.2 poplar genome assembly that was used for short read mapping. Third, the short sizes of the 25 bp Affymetryx oligos used on the array, and sequence differentiation and high heterozygosity within and among species, create further obstacles to making direct comparisons and inferences. Fourth, the *in vitro* culture systems and hormones employed in the current study *vs.* the transcription profiling study differed in some important respects, including use of distinct basal media, and types and/or concentrations of plant hormones used during dedifferentiation and shoot differentiation steps. The extent of callus growth prior to visible shoot regeneration was also, on average, two months longer for *P. trichocarpa* Nisqually-1 compared to *P. tremula* x *alba* 717-1B4, and the frequency of shoot initiation per callus much less. Finally, these *in vitro* systems represent only a subset of the diversity of callogenic-organogenic systems used in poplar and related species. Commonly used systems employ distinct explant tissue types and diverse combinations of growth media, hormones, and environmental conditions. Thus, we believe that expression-5mC associations are likely to be both much stronger and highly diverse, and thus warrant further exploration.

Variation in genome methylation appeared to increase during *in vitro* culture. We found relatively low variance in 5mC among biological replicates from explant and callus tissues. In contrast, 5mC showed extremely high variance in biological replicates of regenerated internodes. This was obvious in hierearchical clustering where biological replicates from regenerated internodes did not cluster together as did replicates from explants and calli. It was also obvious in the much higher variances among biological replications observed for regenerated explants vs. the other tissue types. While some of this variability may be due to the particular tissue culture system employed for this work (with its long period of callus growth prior to shoot regeneration, as discussed above), this finding is consistent with several studies that have reported 5mC polymorphisms among plants regenerated from calli [9-11]. In *Arabidopsis*, clonal regenerants showed a range of mutations including base substitutions, insertions, and deletions that resulted in variant phenotypes [29]. We did not maintain clonal regenerants over a long enough period to be able to observe mutant phenotypes. In addition, each biological replicate was composed of internodes from several regenerated plantlets, and a few individuals may have accounted disproportionately for the variation seen in the sample. Nonetheless, our results support the hypothesis that increased variation in 5mC contributes to the epigenetic instability often observed after *in vitro* regeneration.

Plant cell dedifferentiation involves two distinct processes: acquisition of pluripotency and reentry into the mitotic S phase. These processes are characterized by large changes in gene expression, which in turn are enabled by major changes in chromatin structure, especially chromatin decondensation [4]. Histone methylation may be required for establishment and maintenance of a dedifferentiated cell state via upregulation of genes involved in the ubiquitin proteolytic pathway [30]. Our results support this hypothesis, as analysis of 5mC-enriched genes specific to callus included GO categories related to intracellular protein transport, ubiquitin proteolysis, and protein catabolism.

We found an overall decrease in genome 5mC through callogenesis and redifferentiation. Both promoter and intergenic regions followed this trend. Previous studies of genomic 5mC content in dedifferentiating cells in *Arabidopsis* have reported mixed results [16,17]. In rice, bisulfite genome sequencing showed that DNA methylation was modified in a wide variety of genome regions in both transgenic and *in vitro* regenerated plants [31]. The overall level of 5mC in plant genomes is determined, in large part, by methyltransferase activity. In comparisons of 5mC with expression microarray data, we found that expression of three methyltransferase homologs was higher in callus tissue relative to explants, and still higher in regenerated tissue. Increases in methyltransferase expression coincident with genome-wide hypomethylation have been reported in oil palm calli [32], as well as in many different types of human cancers [33,34], and have been described as an apparent paradox. However, this general trend may belie a great deal of genomic complexity in methylation patterns; as particular genomic features are becoming hypomethylated, others may be hypermethylated. The increased methyltransferase activity may also represent an adaptive response to hypomethylation, even if inadequate to fully recover methylation levels.

Transposable elements (TEs) are abundant features in plant genomes that are frequently marked by cytosine methylation. We examined four major categories of transposable elements from the RepBase transposable element annotation database. *Copia*, *Gypsy* and *Ogre* are long terminal repeat (LTR) retroelements, the predominant type of transposable element sequences in plant genomes [35]. *En/Spm* (CACTA) is one of 19 families classified as eukaryotic “cut-and-paste” DNA TEs [36]. Our results showed that all four TE categories had their highest 5mC content in regenerated tissue. The three retroelement categories increased in 5mC through dedifferentiation and redifferentiation; the EnSpm category did not have significantly increased 5mC in explant and callus stages, but did have a significant increase in 5mC in regenerated internodes. Previous studies using methylation-deficient mutants have demonstrated that hypomethylation can result in transposable element mobilization (Fujomoto et al., 2008). The increased 5mC in TEs in the present study suggests the operation of adaptive genomic mechanisms that might help protect genomes against transposable element spread. It would be of interest to measure whether there is indeed TE element mobilization during *in vitro* culture in future studies.

We found that chromosomal regions with higher gene density underwent gene body hypermethylation in callus tissue that was then released during regeneration. A similar result has been reported for *Arabidopsis*, in which cell suspension cultures exhibited hypermethylation of euchromatin [15]. Bao et al. [25] reported large scale transcriptome reorganization during early callus induction; they catalogued >3,000 poplar genes with at least 5-fold changes in expression during callus induction, with roughly equal numbers of up-regulated and down-regulated genes. When we examined genes in terms of expression deciles, gene body 5mC in callus was higher than in the differentiated tissues in every decile. While gene body 5mC appeared to have a repressive effect on expression in explants and regenerated internodes, the transient body 5mC observed in calli did not appear to repress transcription (Additional file 6). Genes with moderate transcription levels had the highest overall 5mC, similar to what has been observed in *Arabidopsis*[37]. Methylation patterns along plant genes showed minima around transcription start and termination sites, with higher levels over transcribed gene bodies [28,37]. We found sharp increases in gene body methylation in callus tissue, followed by even sharper decreases in redifferentiated internode tissue. This transient gene body methylation was also correlated with higher chromosomal gene density (Figure 8 and 9). Hypermethylation of gene bodies may protect these genes against potential transposable element reactivation from TEs that reside in introns, or possibly from TE insertion into coding regions. In addition, gene body methylation may serve to prevent aberrant transcriptional initiation [1]. In conditions of physiological stress such as during *in vitro* cellular dedifferentiation, gene body methylation may serve as a protective mechanism in chromosomal regions with higher gene density; as euchromatin decondenses, it may become more transcriptionally active and thus more prone to ectopic gene expression.

## Conclusions

Cytosine DNA methylation has long been recognized as a mechanism for genome stabilization, playing a role in control of chromatin dynamics during development, suppressing transposable element activity, and repressing expression of particular genes at specific times during developmental or under specific environmental conditions. Here, we show that the protective role of 5mC is not necessarily at odds with gene expression, and in fact increases in regions of chromosomes where large numbers of genes are undergoing transcriptional upregulation during a developmental fate switch. Our results demonstrate great complexity in epigenomic processes when cells are subject to conditions that induce them to acquire pluripotency and redifferentiate. Given this complexity, it is not surprising that plants regenerated from dedifferentiated cells exhibit a wide variety of epigenomes and attendant phenotypic diversity.

## Methods

### Callus induction and shoot regeneration from Nisqually-1

Micro-cuttings of genotype Nisqually-1 were initially cultured on hormone-free WPM media (Lloyd and McCown 1981). Shoot cultures were maintained on this medium at 25°C under a 16-h photoperiod. Light was provided by fluorescent tubes (TL70, F25T8/TL735, Philips) at a photon flux density of 45 μE · m^-2^ s^-1^.

Three- to five- cm internode explants were collected from *in vitro*-grown, 40- to 50-day old poplar plantlets and cultured on callus induction medium (CIM-NB):MS + F vitamin + 1 mg/L NAA + 1 mg/L BA + Phytagel 1 g/L + Phytablend 1.5 g/L, pH 5.8). These were incubated in the dark for 30 days, and subcultured once on day 14. Resulting calli were then transferred to shoot induction medium (SIM-BN): MS + F vitamin + 0.1 mg/L NAA + 1 mg/L BA + Phytagel 1 g/L + Phytablend 1.5 g/L, pH 5.8) under light and subcultured three times at 3–4 week intervals, until shoots regenerated, ~ 4 months. Regenerated shoots were propagated on WPM until roots grew and shoots were substantial enough for sample collection, ~ 2 months. Regenerated internodes were collected at a single time point. Each biological replicate for internode explants and regenerated internodes consisted of tissue from 5–6 magenta boxes (25–30 shoots).

### Molecular methods

Total genomic DNA was isolated from three biological replicates of each tissue stage using a CTAB-based method, and the methylated DNA fraction was immunoprecipitated with an antibody to 5-methylcytidine, as described previously [28]. A non-immunoprecipitated sample was sequenced as a control.

### Bioinformatic analyses

Illumina read trimming, filtering, normalization, and alignment to the *P. trichocarpa* V2.2 reference genome sequence were conducted as described previously [28]. Reads per 1 kb target sequence per million reads mapped (RPKM) were calculated for 1 kb tiled genome windows and for genomic features (gene promoters, gene bodies, 5’ and 3’ UTRs, intergenic regions). To determine whether genome windows and genomic features were 5mC-enriched relative to the non-immunoprecipitated control, we modeled the counts of the sequencing reads using negative binomial distributions. A negative binomial distribution uses a dispersion parameter to capture the extra-Poisson variation that is often observed in sequencing read counts from independent biological samples. It has been observed in this study and in earlier RNA-Seq studies that the amount of dispersion often depends on 5mC or expression level, [38,39]. We modeled the dispersion parameter as a smooth function of the mean relative frequency of the sequencing counts and fit a separate dispersion model to each group of plants. For comparing 5mC levels of features between two groups, we fit a negative binomial regression model to each feature using the indicator of group membership as a predicting variable, and identified differentially-5mC-enriched features by testing the corresponding regression coefficient. We computed test p-values using a likelihood ratio test with high-order asymptotic adjustment.

Basic data manipulations and statistical tests were performed using the R base package (http://www.r-project.org/). A Pearson’s product–moment correlation test was run to determine overall correlation between gene density and 5mC within each tissue, and over all tissues, using all biological replicates. To further examine the relationship between gene density and gene body 5mC, genes were binned in five gene density groups from low to high, and paired t-tests with average MeDIP-seq read countsover genes within each binwere used to make pairwise comparisons within each bin (explant *vs.* callus, explant *vs.* regen, callus *vs.* regen). Gene expression-5mC relationships were examined by filtering the set of genes with > =2-fold expression differences, and the 27,753 that could be cross-referenced, from available Affymetrix microarray data (Bao et al., 2009), and running pairwise t-tests on gene RPKM for each pair of tissues, assuming independence of gene values within biological replicates.Gene Ontology (GO) category enrichment for selected gene sets was determined using the AgriGO Singular Enrichment Analysis (SEA) tool (http://bioinfo.cau.edu.cn/agriGO/analysis.php), using the Fisher’s Exact Test statistical method with Benjamini and Hochberg multi-test correction option. RPKM means and variances for the sets of 1 kb tiled genome windows were calculated among biological replicates within each tissue.

## Competing interests

The authors declare no competing interests.

## Authors’ contributions

SHS, TM and MF designed the experiment. KJV, KRP, and CM performed the research. LJW, MP and YD performed bioinformatic analyses. KJV, KRP, MF and SHS wrote the paper. All authors read and approved the final manuscript.

## Supplementary Material

Additional file 1 **Clustering of biological replicates based on RPKM values of gene bodies.** Hierarchical clustering of biological replicates from all tissues. Distance matrices were based on Pearson correlation of RPKM values for all annotated gene bodies in the *P. trichocarpa* V 2.2 reference genome. Click here for file

Additional file 2 **Gene body 5mC shows a transient increase in dedifferentiated tissue.** Boxplots showing RPKM averaged over all genes using all biological replicates within each tissue. **A**. Outliers not shown. **B**. Outliers shown. Click here for file

Additional file 3 **Overrepresented gene ontology (GO) terms associated with body-methylated genes in callus tissue.** GO analysis was performed with the AgriGo Singular Enrichment Analysis tool (http://bioinfo.cau.edu.cn/agriGO/analysis.php). Click here for file

Additional file 4 **Relationship of gene expression to DNA methylation among *in vitro *tissue types.** Genes were divided into deciles from low to high expression level, and gene body RPKMs for each decile plotted. Click here for file

Additional file 5 **Relationship of gene expression to gene body DNA methylation among *in vitro *tissue types.** Genes were divided into deciles from low to high expression level, and gene body median RPKM for each decile was plotted Numbers of genes per decile were 7,534-7,843 in decile 1, 2,077-3,781 in deciles 2–6, 1,749-1936 in decile 7, 334–947 in deciles 8 and 9, and 36–90 in decile 10. Click here for file

Additional file 6 **Relationship of gene feature methylation to expression.** The logarithm of expression for significantly (Q < 0.10) methylated vs. unmethylated gene-associated features is shown in relation to in vitro tissue type. Click here for file

Additional file 7 **Changes in gene expression for homologs of *Arabidopsis *establishment and maintenance methyltransferases during *in vitro *regeneration.** Of nine homologs of Arabidopsis DNA methyltransferases (MET1/2), CHROMOMETHYLASE 3 (CMT3), DECREASE IN DNA METHYLATION 1 (DDM1), and DOMAINS REARRANGED METHYLTRANSFERASE 1 and 2 (DRM1/2), only the three shown had greater than 1.5-fold changes in expression during dedifferentiation. Click here for file
