# Supplementary figures and images for: Methylome reorganization during in vitro dedifferentiation and regeneration of Populus trichocarpa
Source: BMC Plant Biol. 2013 Jun 25;13:92. doi: 10.1186/1471-2229-13-92 (PMC3728041; doi:10.1186/1471-2229-13-92)

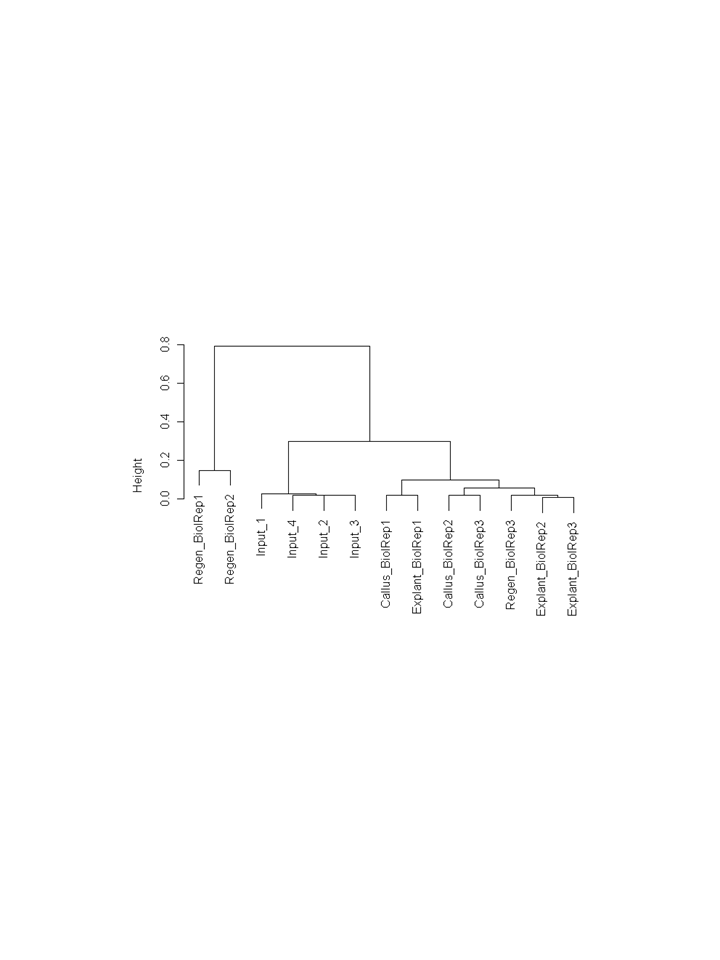

Supplement: Additional file 1 — Clustering of biological replicates based on RPKM values of gene bodies. Hierarchical clustering of biological replicates from all tissues. Distance matrices were based on Pearson correlation of RPKM values for all annotated gene bodies in the P. trichocarpa V 2.2 reference genome. [file 1471-2229-13-92-S1.tiff]

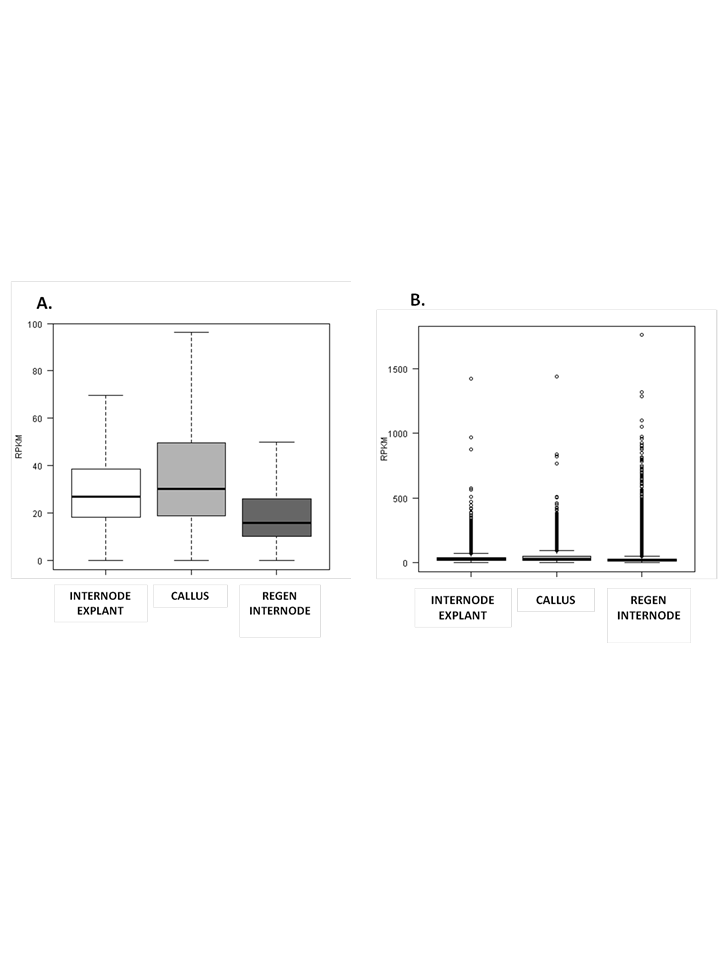

Supplement: Additional file 2 — Gene body 5mC shows a transient increase in dedifferentiated tissue. Boxplots showing RPKM averaged over all genes using all biological replicates within each tissue. A. Outliers not shown. B. Outliers shown. [file 1471-2229-13-92-S2.tiff]

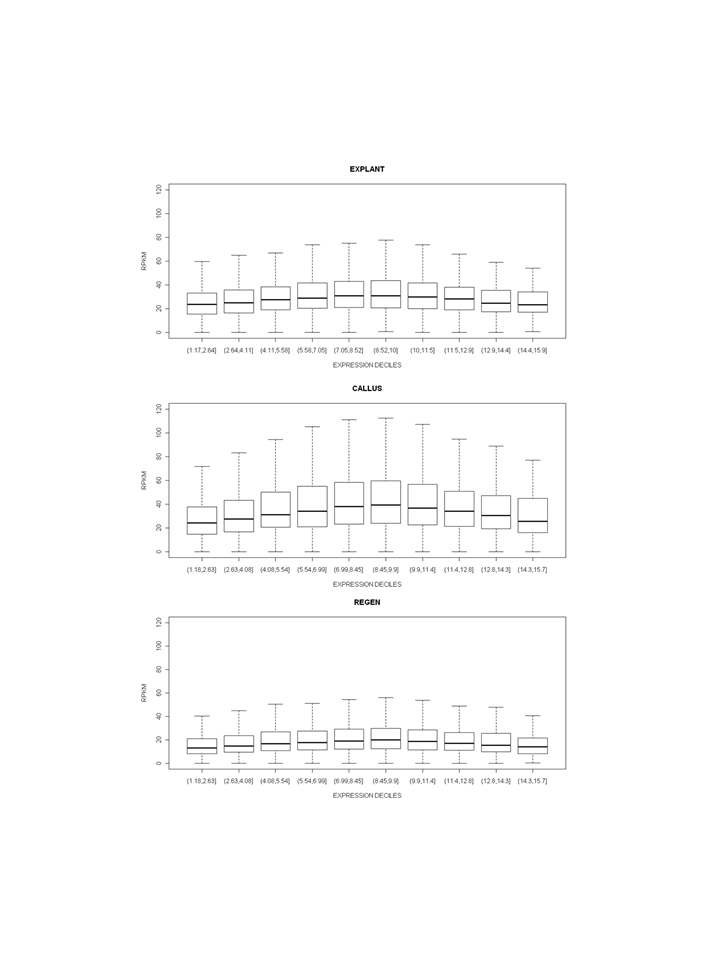

Supplement: Additional file 4 — Relationship of gene expression to DNA methylation among in vitro tissue types. Genes were divided into deciles from low to high expression level, and gene body RPKMs for each decile plotted. [file 1471-2229-13-92-S4.tiff]

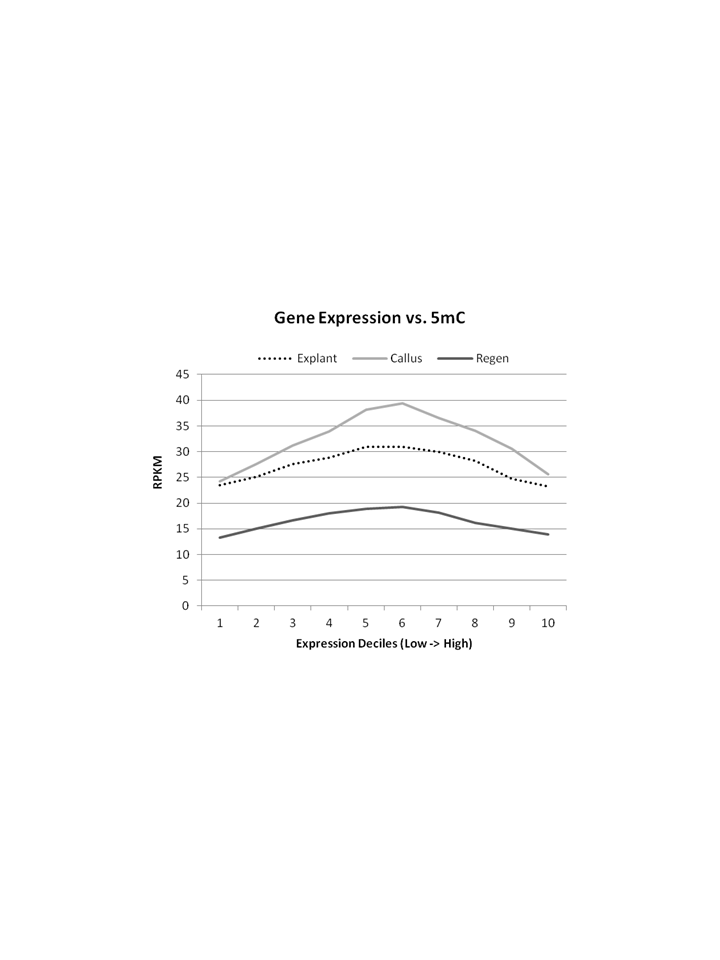

Supplement: Additional file 5 — Relationship of gene expression to gene body DNA methylation among in vitro tissue types. Genes were divided into deciles from low to high expression level, and gene body median RPKM for each decile was plotted Numbers of genes per decile were 7,534-7,843 in decile 1, 2,077-3,781 in deciles 2–6, 1,749-1936 in decile 7, 334–947 in deciles 8 and 9, and 36–90 in decile 10. [file 1471-2229-13-92-S5.tiff]

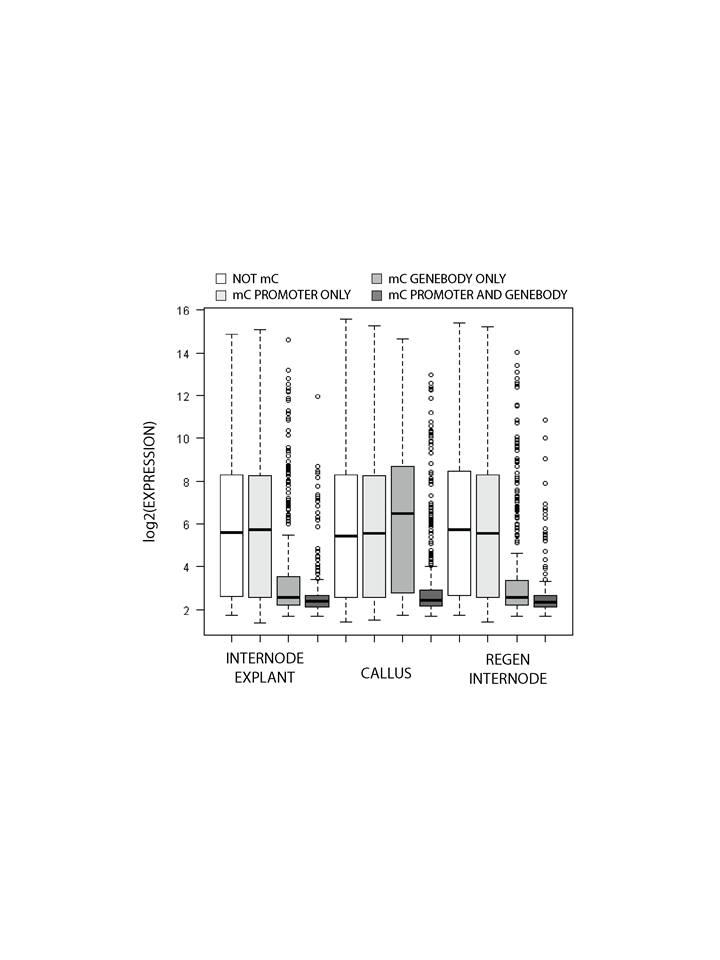

Supplement: Additional file 6 — Relationship of gene feature methylation to expression. The logarithm of expression for significantly (Q < 0.10) methylated vs. unmethylated gene-associated features is shown in relation to in vitro tissue type. [file 1471-2229-13-92-S6.tiff]

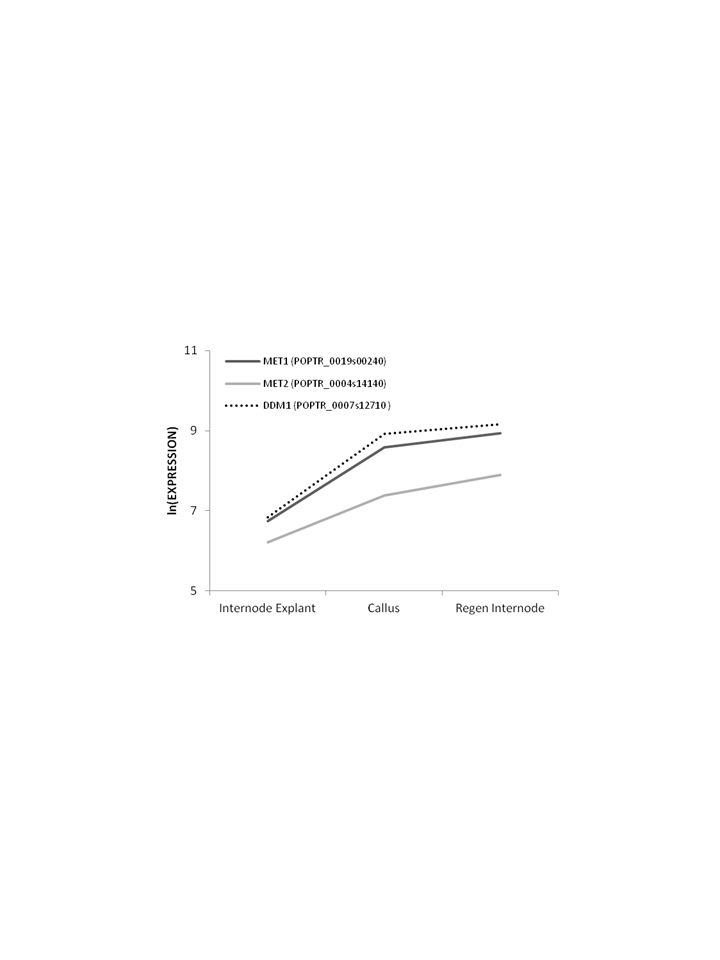

Supplement: Additional file 7 — Changes in gene expression for homologs of Arabidopsis establishment and maintenance methyltransferases during in vitro regeneration. Of nine homologs of Arabidopsis DNA methyltransferases (MET1/2), CHROMOMETHYLASE 3 (CMT3), DECREASE IN DNA METHYLATION 1 (DDM1), and DOMAINS REARRANGED METHYLTRANSFERASE 1 and 2 (DRM1/2), only the three shown had greater than 1.5-fold changes in expression during dedifferentiation. [file 1471-2229-13-92-S7.tiff]
