# Supplementary material for: Methylome reorganization during in vitro dedifferentiation and regeneration of Populus trichocarpa
Source: BMC Plant Biol. 2013 Jun 25;13:92. doi: 10.1186/1471-2229-13-92 (PMC3728041; doi:10.1186/1471-2229-13-92)

| **GO term** | **Ontology** | **Description** | **Number in input list** | **Number in BG/Ref** | **p-value** | **FDR** |
| --- | --- | --- | --- | --- | --- | --- |
| GO:0051641 | P | cellular localization | 43 | 306 | 1.50E-13 | 3.20E-11 |
| GO:0006886 | P | intracellular protein transport | 32 | 187 | 8.10E-13 | 1.70E-10 |
| GO:0070727 | P | cellular macromolecule localization | 33 | 199 | 8.90E-13 | 1.90E-10 |
| GO:0034613 | P | cellular protein localization | 33 | 199 | 8.90E-13 | 1.90E-10 |
| GO:0051649 | P | establishment of localization in cell | 40 | 285 | 1.10E-12 | 2.20E-10 |
| GO:0008104 | P | protein localization | 40 | 288 | 1.50E-12 | 3.10E-10 |
| GO:0009987 | P | cellular process | 458 | 8559 | 4.40E-12 | 9.30E-10 |
| GO:0033036 | P | macromolecule localization | 40 | 300 | 5.40E-12 | 1.10E-09 |
| GO:0046907 | P | intracellular transport | 34 | 225 | 6.00E-12 | 1.30E-09 |
| GO:0044281 | P | small molecule metabolic process | 74 | 839 | 2.80E-11 | 5.70E-09 |
| GO:0045184 | P | establishment of protein localization | 35 | 265 | 1.40E-10 | 2.80E-08 |
| GO:0015031 | P | protein transport | 35 | 265 | 1.40E-10 | 2.80E-08 |
| GO:0006807 | P | nitrogen compound metabolic process | 182 | 2940 | 2.20E-10 | 4.50E-08 |
| GO:0006418 | P | tRNA aminoacylation for protein translation | 17 | 68 | 3.80E-10 | 7.50E-08 |
| GO:0043038 | P | amino acid activation | 17 | 68 | 3.80E-10 | 7.50E-08 |
| GO:0043039 | P | tRNA aminoacylation | 17 | 68 | 3.80E-10 | 7.50E-08 |
| GO:0006139 | P | nucleobase, nucleoside, nucleotide and nucleic acid metabolic process | 164 | 2598 | 3.80E-10 | 7.50E-08 |
| GO:0034660 | P | ncRNA metabolic process | 24 | 141 | 5.90E-10 | 1.20E-07 |
| GO:0006399 | P | tRNA metabolic process | 20 | 108 | 3.30E-09 | 6.60E-07 |
| GO:0016192 | P | vesicle-mediated transport | 26 | 200 | 4.00E-08 | 7.90E-06 |
| GO:0044237 | P | cellular metabolic process | 342 | 6668 | 4.40E-08 | 8.50E-06 |
| GO:0044238 | P | primary metabolic process | 376 | 7479 | 8.10E-08 | 1.60E-05 |
| GO:0051056 | P | regulation of small GTPase mediated signal transduction | 13 | 63 | 4.80E-07 | 9.20E-05 |
| GO:0046578 | P | regulation of Ras protein signal transduction | 13 | 63 | 4.80E-07 | 9.20E-05 |
| GO:0007265 | P | Ras protein signal transduction | 13 | 63 | 4.80E-07 | 9.20E-05 |
| GO:0055086 | P | nucleobase, nucleoside and nucleotide metabolic process | 28 | 256 | 4.80E-07 | 9.20E-05 |
| GO:0008152 | P | metabolic process | 461 | 9587 | 5.20E-07 | 9.80E-05 |
| GO:0006259 | P | DNA metabolic process | 25 | 214 | 5.60E-07 | 0.00011 |
| GO:0006520 | P | cellular amino acid metabolic process | 27 | 247 | 7.70E-07 | 0.00014 |
| GO:0009117 | P | nucleotide metabolic process | 25 | 218 | 8.00E-07 | 0.00015 |
| GO:0006753 | P | nucleoside phosphate metabolic process | 25 | 218 | 8.00E-07 | 0.00015 |
| GO:0009966 | P | regulation of signal transduction | 13 | 67 | 1.00E-06 | 0.00018 |
| GO:0023051 | P | regulation of signaling process | 13 | 67 | 1.00E-06 | 0.00018 |
| GO:0010646 | P | regulation of cell communication | 13 | 67 | 1.00E-06 | 0.00018 |
| GO:0034641 | P | cellular nitrogen compound metabolic process | 35 | 380 | 1.30E-06 | 0.00023 |
| GO:0016070 | P | RNA metabolic process | 87 | 1366 | 2.10E-06 | 0.00037 |
| GO:0009308 | P | amine metabolic process | 30 | 308 | 2.20E-06 | 0.0004 |
| GO:0044106 | P | cellular amine metabolic process | 27 | 264 | 2.80E-06 | 0.00049 |
| GO:0006396 | P | RNA processing | 22 | 190 | 3.00E-06 | 0.00053 |
| GO:0009152 | P | purine ribonucleotide biosynthetic process | 19 | 149 | 3.30E-06 | 0.00058 |
| GO:0009150 | P | purine ribonucleotide metabolic process | 19 | 149 | 3.30E-06 | 0.00058 |
| GO:0009260 | P | ribonucleotide biosynthetic process | 19 | 152 | 4.40E-06 | 0.00077 |
| GO:0009259 | P | ribonucleotide metabolic process | 19 | 152 | 4.40E-06 | 0.00077 |
| GO:0044265 | P | cellular macromolecule catabolic process | 24 | 227 | 5.40E-06 | 0.00093 |
| GO:0043170 | P | macromolecule metabolic process | 293 | 5955 | 6.00E-06 | 0.001 |
| GO:0043436 | P | oxoacid metabolic process | 33 | 375 | 6.50E-06 | 0.0011 |
| GO:0019752 | P | carboxylic acid metabolic process | 33 | 375 | 6.50E-06 | 0.0011 |
| GO:0030163 | P | protein catabolic process | 19 | 156 | 6.50E-06 | 0.0011 |
| GO:0006082 | P | organic acid metabolic process | 33 | 376 | 6.80E-06 | 0.0011 |
| GO:0006163 | P | purine nucleotide metabolic process | 19 | 157 | 7.20E-06 | 0.0012 |
| GO:0006519 | P | cellular amino acid and derivative metabolic process | 27 | 278 | 7.20E-06 | 0.0012 |
| GO:0009057 | P | macromolecule catabolic process | 27 | 278 | 7.20E-06 | 0.0012 |
| GO:0042180 | P | cellular ketone metabolic process | 33 | 377 | 7.20E-06 | 0.0012 |
| GO:0006164 | P | purine nucleotide biosynthetic process | 19 | 157 | 7.20E-06 | 0.0012 |
| GO:0006511 | P | ubiquitin-dependent protein catabolic process | 17 | 130 | 7.50E-06 | 0.0012 |
| GO:0043632 | P | modification-dependent macromolecule catabolic process | 17 | 130 | 7.50E-06 | 0.0012 |
| GO:0019941 | P | modification-dependent protein catabolic process | 17 | 130 | 7.50E-06 | 0.0012 |
| GO:0009165 | P | nucleotide biosynthetic process | 20 | 174 | 9.10E-06 | 0.0014 |
| GO:0051603 | P | proteolysis involved in cellular protein catabolic process | 18 | 147 | 1.00E-05 | 0.0016 |
| GO:0044257 | P | cellular protein catabolic process | 18 | 147 | 1.00E-05 | 0.0016 |
| GO:0044260 | P | cellular macromolecule metabolic process | 268 | 5448 | 1.30E-05 | 0.002 |
| GO:0044248 | P | cellular catabolic process | 25 | 261 | 1.90E-05 | 0.003 |
| GO:0032318 | P | regulation of Ras GTPase activity | 10 | 53 | 2.30E-05 | 0.0035 |
| GO:0043087 | P | regulation of GTPase activity | 10 | 53 | 2.30E-05 | 0.0035 |
| GO:0007017 | P | microtubule-based process | 15 | 116 | 2.90E-05 | 0.0043 |
| GO:0051336 | P | regulation of hydrolase activity | 10 | 55 | 3.20E-05 | 0.0048 |
| GO:0006996 | P | organelle organization | 21 | 207 | 3.70E-05 | 0.0055 |
| GO:0007018 | P | microtubule-based movement | 13 | 95 | 5.30E-05 | 0.0078 |
| GO:0051179 | P | localization | 86 | 1474 | 5.30E-05 | 0.0078 |
| GO:0046034 | P | ATP metabolic process | 15 | 129 | 9.90E-05 | 0.014 |
| GO:0006754 | P | ATP biosynthetic process | 15 | 129 | 9.90E-05 | 0.014 |
| GO:0009056 | P | catabolic process | 29 | 359 | 0.0001 | 0.015 |
| GO:0051276 | P | chromosome organization | 13 | 103 | 0.00012 | 0.017 |
| GO:0009199 | P | ribonucleoside triphosphate metabolic process | 15 | 137 | 0.00019 | 0.026 |
| GO:0009205 | P | purine ribonucleoside triphosphate metabolic process | 15 | 137 | 0.00019 | 0.026 |
| GO:0009201 | P | ribonucleoside triphosphate biosynthetic process | 15 | 137 | 0.00019 | 0.026 |
| GO:0009142 | P | nucleoside triphosphate biosynthetic process | 15 | 137 | 0.00019 | 0.026 |
| GO:0009144 | P | purine nucleoside triphosphate metabolic process | 15 | 137 | 0.00019 | 0.026 |
| GO:0009145 | P | purine nucleoside triphosphate biosynthetic process | 15 | 137 | 0.00019 | 0.026 |
| GO:0009206 | P | purine ribonucleoside triphosphate biosynthetic process | 15 | 137 | 0.00019 | 0.026 |
| GO:0032011 | P | ARF protein signal transduction | 7 | 34 | 0.00022 | 0.029 |
| GO:0032012 | P | regulation of ARF protein signal transduction | 7 | 34 | 0.00022 | 0.029 |
| GO:0009141 | P | nucleoside triphosphate metabolic process | 15 | 139 | 0.00023 | 0.03 |
| GO:0048278 | P | vesicle docking | 6 | 25 | 0.00025 | 0.033 |
| GO:0022406 | P | membrane docking | 6 | 25 | 0.00025 | 0.033 |
| GO:0006810 | P | transport | 81 | 1450 | 0.00032 | 0.041 |
| GO:0051234 | P | establishment of localization | 81 | 1450 | 0.00032 | 0.041 |
| GO:0016032 | P | viral reproduction | 6 | 26 | 0.00032 | 0.041 |
| GO:0046483 | P | heterocycle metabolic process | 24 | 297 | 0.00038 | 0.048 |
| GO:0016817 | F | hydrolase activity, acting on acid anhydrides | 111 | 890 | 2.40E-27 | 3.50E-25 |
| GO:0017111 | F | nucleoside-triphosphatase activity | 103 | 810 | 3.30E-26 | 4.80E-24 |
| GO:0016818 | F | hydrolase activity, acting on acid anhydrides, in phosphorus-containing anhydrides | 105 | 849 | 1.00E-25 | 1.50E-23 |
| GO:0016462 | F | pyrophosphatase activity | 103 | 823 | 1.10E-25 | 1.60E-23 |
| GO:0003676 | F | nucleic acid binding | 241 | 3300 | 1.30E-20 | 1.90E-18 |
| GO:0005488 | F | binding | 605 | 10853 | 1.30E-17 | 1.80E-15 |
| GO:0004386 | F | helicase activity | 40 | 223 | 2.50E-16 | 3.50E-14 |
| GO:0000166 | F | nucleotide binding | 264 | 4088 | 7.20E-16 | 1.00E-13 |
| GO:0017076 | F | purine nucleotide binding | 257 | 3957 | 8.50E-16 | 1.20E-13 |
| GO:0032555 | F | purine ribonucleotide binding | 246 | 3761 | 1.40E-15 | 2.00E-13 |
| GO:0032553 | F | ribonucleotide binding | 246 | 3761 | 1.40E-15 | 2.00E-13 |
| GO:0016787 | F | hydrolase activity | 194 | 2834 | 1.40E-14 | 1.90E-12 |
| GO:0003723 | F | RNA binding | 55 | 445 | 2.00E-14 | 2.70E-12 |
| GO:0001883 | F | purine nucleoside binding | 231 | 3621 | 9.60E-14 | 1.30E-11 |
| GO:0030554 | F | adenyl nucleotide binding | 231 | 3621 | 9.60E-14 | 1.30E-11 |
| GO:0001882 | F | nucleoside binding | 231 | 3622 | 9.90E-14 | 1.30E-11 |
| GO:0032559 | F | adenyl ribonucleotide binding | 221 | 3432 | 1.20E-13 | 1.60E-11 |
| GO:0005524 | F | ATP binding | 221 | 3432 | 1.20E-13 | 1.60E-11 |
| GO:0016887 | F | ATPase activity | 49 | 459 | 9.20E-11 | 1.20E-08 |
| GO:0004812 | F | aminoacyl-tRNA ligase activity | 17 | 69 | 4.80E-10 | 6.00E-08 |
| GO:0016875 | F | ligase activity, forming carbon-oxygen bonds | 17 | 69 | 4.80E-10 | 6.00E-08 |
| GO:0016876 | F | ligase activity, forming aminoacyl-tRNA and related compounds | 17 | 69 | 4.80E-10 | 6.00E-08 |
| GO:0003824 | F | catalytic activity | 472 | 9307 | 1.60E-09 | 2.00E-07 |
| GO:0008270 | F | zinc ion binding | 92 | 1259 | 2.60E-09 | 3.20E-07 |
| GO:0008026 | F | ATP-dependent helicase activity | 19 | 116 | 6.40E-08 | 7.80E-06 |
| GO:0070035 | F | purine NTP-dependent helicase activity | 19 | 116 | 6.40E-08 | 7.80E-06 |
| GO:0016874 | F | ligase activity | 36 | 357 | 1.00E-07 | 1.20E-05 |
| GO:0003774 | F | motor activity | 19 | 125 | 2.20E-07 | 2.60E-05 |
| GO:0042623 | F | ATPase activity, coupled | 28 | 279 | 2.70E-06 | 0.00031 |
| GO:0005096 | F | GTPase activator activity | 10 | 53 | 2.30E-05 | 0.0027 |
| GO:0005083 | F | small GTPase regulator activity | 13 | 88 | 2.30E-05 | 0.0027 |
| GO:0060589 | F | nucleoside-triphosphatase regulator activity | 14 | 103 | 3.00E-05 | 0.0034 |
| GO:0030695 | F | GTPase regulator activity | 13 | 91 | 3.30E-05 | 0.0038 |
| GO:0031625 | F | ubiquitin protein ligase binding | 5 | 12 | 4.50E-05 | 0.0051 |
| GO:0008047 | F | enzyme activator activity | 10 | 60 | 6.90E-05 | 0.0078 |
| GO:0008565 | F | protein transporter activity | 11 | 77 | 0.00013 | 0.015 |
| GO:0003924 | F | GTPase activity | 14 | 119 | 0.00015 | 0.016 |
| GO:0004518 | F | nuclease activity | 16 | 149 | 0.00015 | 0.017 |
| GO:0019899 | F | enzyme binding | 5 | 15 | 0.00016 | 0.017 |
| GO:0003777 | F | microtubule motor activity | 13 | 107 | 0.00018 | 0.019 |
| GO:0019001 | F | guanyl nucleotide binding | 26 | 336 | 0.00043 | 0.046 |
| GO:0005622 | C | intracellular | 236 | 3314 | 5.30E-19 | 3.20E-17 |
| GO:0044424 | C | intracellular part | 180 | 2492 | 1.00E-15 | 6.00E-14 |
| GO:0044464 | C | cell part | 321 | 5684 | 1.10E-11 | 6.20E-10 |
| GO:0005623 | C | cell | 321 | 5684 | 1.10E-11 | 6.20E-10 |
| GO:0048475 | C | coated membrane | 16 | 64 | 1.20E-09 | 6.70E-08 |
| GO:0030117 | C | membrane coat | 16 | 64 | 1.20E-09 | 6.70E-08 |
| GO:0043231 | C | intracellular membrane-bounded organelle | 95 | 1342 | 6.90E-09 | 3.70E-07 |
| GO:0005634 | C | nucleus | 75 | 975 | 8.10E-09 | 4.30E-07 |
| GO:0043234 | C | protein complex | 56 | 646 | 1.00E-08 | 5.30E-07 |
| GO:0043227 | C | membrane-bounded organelle | 95 | 1354 | 1.00E-08 | 5.30E-07 |
| GO:0043229 | C | intracellular organelle | 118 | 1910 | 2.10E-07 | 1.00E-05 |
| GO:0043226 | C | organelle | 118 | 1910 | 2.10E-07 | 1.00E-05 |
| GO:0030118 | C | clathrin coat | 8 | 21 | 4.80E-07 | 2.30E-05 |
| GO:0030119 | C | AP-type membrane coat adaptor complex | 7 | 17 | 1.40E-06 | 6.30E-05 |
| GO:0030131 | C | clathrin adaptor complex | 7 | 17 | 1.40E-06 | 6.30E-05 |
| GO:0005737 | C | cytoplasm | 76 | 1165 | 3.60E-06 | 0.00016 |
| GO:0005694 | C | chromosome | 13 | 84 | 1.40E-05 | 0.00061 |
| GO:0044422 | C | organelle part | 36 | 446 | 1.70E-05 | 0.00072 |
| GO:0044446 | C | intracellular organelle part | 36 | 446 | 1.70E-05 | 0.00072 |
| GO:0016459 | C | myosin complex | 6 | 17 | 2.30E-05 | 0.00094 |
| GO:0016023 | C | cytoplasmic membrane-bounded vesicle | 7 | 27 | 4.50E-05 | 0.0016 |
| GO:0031461 | C | cullin-RING ubiquitin ligase complex | 5 | 12 | 4.50E-05 | 0.0016 |
| GO:0030658 | C | transport vesicle membrane | 6 | 19 | 4.70E-05 | 0.0016 |
| GO:0031982 | C | vesicle | 7 | 27 | 4.50E-05 | 0.0016 |
| GO:0031988 | C | membrane-bounded vesicle | 7 | 27 | 4.50E-05 | 0.0016 |
| GO:0031410 | C | cytoplasmic vesicle | 7 | 27 | 4.50E-05 | 0.0016 |
| GO:0030133 | C | transport vesicle | 6 | 19 | 4.70E-05 | 0.0016 |
| GO:0044430 | C | cytoskeletal part | 8 | 40 | 9.70E-05 | 0.0032 |
| GO:0032991 | C | macromolecular complex | 69 | 1145 | 0.0001 | 0.0033 |
| GO:0012505 | C | endomembrane system | 14 | 119 | 0.00015 | 0.0044 |
| GO:0012507 | C | ER to Golgi transport vesicle membrane | 5 | 15 | 0.00016 | 0.0044 |
| GO:0030127 | C | COPII vesicle coat | 5 | 15 | 0.00016 | 0.0044 |
| GO:0030134 | C | ER to Golgi transport vesicle | 5 | 15 | 0.00016 | 0.0044 |
| GO:0030120 | C | vesicle coat | 6 | 25 | 0.00025 | 0.0051 |
| GO:0044433 | C | cytoplasmic vesicle part | 6 | 25 | 0.00025 | 0.0051 |
| GO:0044431 | C | Golgi apparatus part | 9 | 56 | 0.00021 | 0.0051 |
| GO:0030662 | C | coated vesicle membrane | 6 | 25 | 0.00025 | 0.0051 |
| GO:0012506 | C | vesicle membrane | 6 | 25 | 0.00025 | 0.0051 |
| GO:0030659 | C | cytoplasmic vesicle membrane | 6 | 25 | 0.00025 | 0.0051 |
| GO:0015629 | C | actin cytoskeleton | 6 | 25 | 0.00025 | 0.0051 |
| GO:0030135 | C | coated vesicle | 6 | 25 | 0.00025 | 0.0051 |
| GO:0005794 | C | Golgi apparatus | 9 | 72 | 0.0014 | 0.026 |
| GO:0005856 | C | cytoskeleton | 9 | 78 | 0.0024 | 0.044 |


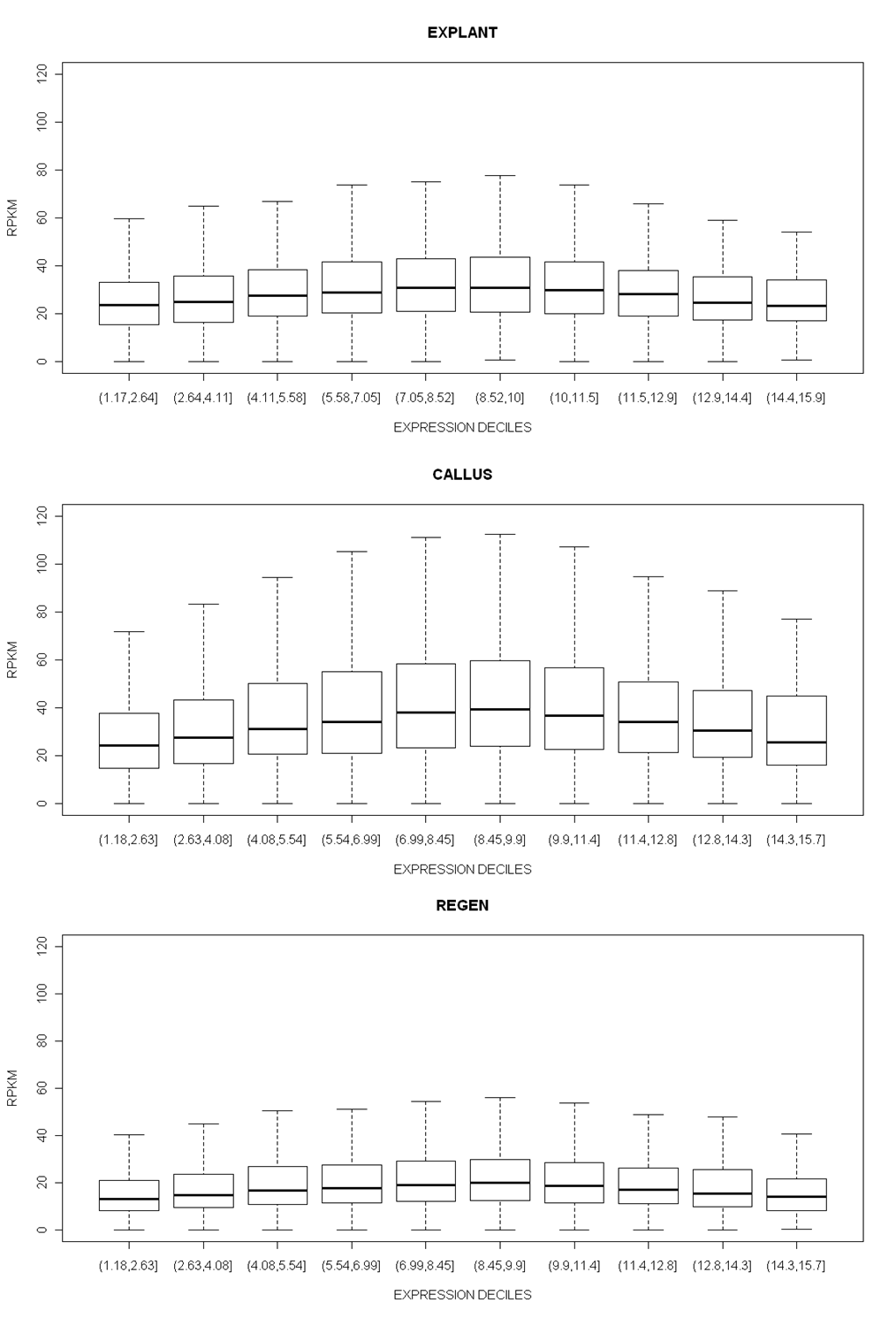

Supplement: Additional file 3 — Overrepresented gene ontology (GO) terms associated with body-methylated genes in callus tissue. GO analysis was performed with the AgriGo Singular Enrichment Analysis tool (http://bioinfo.cau.edu.cn/agriGO/analysis.php). [file 1471-2229-13-92-S3.docx]
